# Synthesis of 10-*O*-aryl-substituted berberine derivatives by Chan–Evans–Lam coupling and investigation of their DNA-binding properties

**DOI:** 10.3762/bjoc.17.81

**Published:** 2021-05-04

**Authors:** Peter Jonas Wickhorst, Mathilda Blachnik, Denisa Lagumdzija, Heiko Ihmels

**Affiliations:** 1 Department of Chemistry – Biology, University of Siegen, and Center of Micro- and Nanochemistry and Engineering (Cµ), Adolf-Reichwein-Str. 2, 57068 Siegen, Germany

**Keywords:** berberine alkaloids, Cu-mediated coupling reactions, DNA recognition, nucleic acids, quadruplex DNA

## Abstract

Eleven novel 10-*O*-aryl-substituted berberrubine and berberine derivatives were synthesized by the Cu^2+^-catalyzed Chan–Evans–Lam coupling of berberrubine with arylboronic acids and subsequent 9-*O*-methylation. The reaction is likely introduced by the Cu^2+^-induced demethylation of berberrubine and subsequent arylation of the resulting 10-oxyanion functionality. Thus, this synthetic route represents the first successful Cu-mediated coupling reaction of berberine substrates. The DNA-binding properties of the 10-*O*-arylberberine derivatives with duplex and quadruplex DNA were studied by thermal DNA denaturation experiments, spectrometric titrations as well as CD and LD spectroscopy. Fluorimetric DNA melting analysis with different types of quadruplex DNA revealed a moderate stabilization of the telomeric quadruplex-forming oligonucleotide sequence G_3_(TTAG_3_)_3_. The derivatives showed a moderate affinity towards quadruplex DNA (*K*_b_ = 5–9 × 10^5^ M^−1^) and ct DNA (*K*_b_ = 3–5 × 10^4^ M^−1^) and exhibited a fluorescence light-up effect upon complexation to both DNA forms, with slightly higher intensity in the presence of the quadruplex DNA. Furthermore, the CD- and LD-spectroscopic studies revealed that the title compounds intercalate into ct DNA and bind to G4-DNA by terminal stacking.

## Introduction

**Figure 1:**
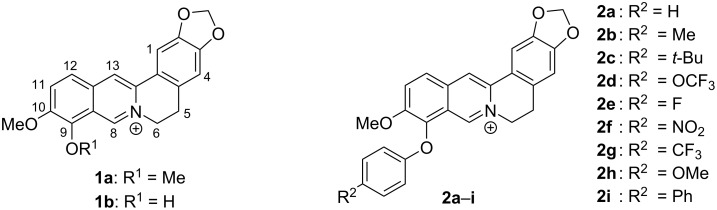
Structures and numbering of berberine (**1a**), berberrubine (**1b**) and 9-*O*-aryl-substituted berberine derivatives **2a**–**i**.

Berberine (**1a**) is the most prominent member of the protoberberine family, i.e., a group of tetracyclic isoquinolinium alkaloids [1]. Like many members of this family, berberine (**1a**) is a natural product and may be isolated from different plants such as *berberis vulgaris*, *hydrastis canadensis* or *coptidis rhizome* [2]. Particularly, the latter is established in traditional Chinese medicine as source for anti-inflammatory extracts [2]. Berberine has also been employed in modern medicine because of its antimicrobial [3], antiprotozoal [4], antiviral [5], and anti-inflammatory [6] activity, and it is used in the treatment of tuberculosis [7], diarrhea [8], diabetes [9], cardiovascular diseases [10] or high cholesterol levels [11]. Most interestingly, it has been found that berberine (**1a**) exhibits a selective cytotoxicity against cancer cells [12–13], which is mainly based on its DNA-binding properties [14]. Since the complexation with DNA leads to significant changes of the fluorescent quantum yields of the bound berberine, it can also be used as fluorescent probe in bioanalytical chemistry [15–16]. Because of these attractive properties of this lead structure, numerous berberine derivatives have been synthesized in order to further improve the biological activity [17–26]. Among those derivatives, specifically 13- and 9-substituted berberine derivatives have gained much interest as selective DNA ligands that specifically target quadruplex DNA (G4-DNA) [23–29], i.e., non-canonical, biologically relevant DNA structures that are formed by the association of four guanine-rich DNA strands [30]. Due to their straightforward synthetic accessibility by substitution at the 9-hydroxy functionality of berberrubine (**1b**), 9-*O*-substituted berberine derivatives are particularly attractive. But so far, only functionalization at this position by alkylation [23–26] and nucleophilic substitution [17–21] of berberrubine (**1b**) have been performed, whereas the direct *O*-arylation of berberrubine has not been accomplished, yet, most likely because the substrate is not fully compatible with the usual conditions of the corresponding Ullmann or Buchwald–Hartwig coupling reactions. Nevertheless, it has been recently reported that 9-*O*-aryl-substituted berberine derivatives can be isolated by an Ullmann-type arylation of a tetrahydroberberrubine and a subsequent oxidation of the primary product to the respective berberine derivatives **2a**–**i** (Figure 1) [31].

So far, a successful direct arylation of berberrubine (**1b**) has not been reported. At the same time, the Chan–Evans–Lam cross coupling has been established as a useful and relatively mild method for metal-mediated arylation of hydroxyarenes [32–33]. Therefore, we proposed that this method may be suitable for the arylation of berberrubine. And herein, we report the application of this Cu-mediated coupling reaction for the synthesis of 10-*O*-arylated berberine derivatives as unexpected reaction product, along with first experiments that demonstrate the G4-DNA-binding properties of this class of berberine derivatives.

## Results and Discussion

### Synthesis

**Scheme 1:**
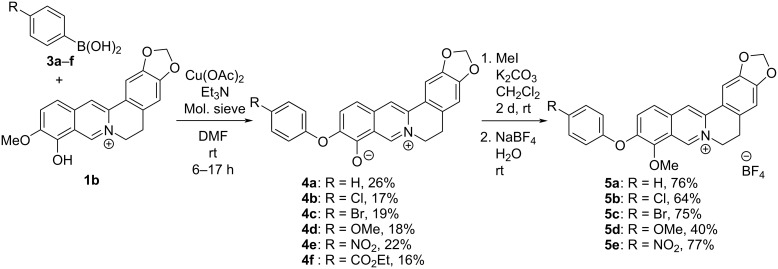
Synthesis of 10-*O*-arylated berberine derivatives **5a**–**e**.

In first orienting experiments, the reaction of berberrubine (**1b**) and phenylboronic acid (**3a**) was performed under typical conditions [34] of a Chan–Evans–Lam coupling with Cu(OAc)_2_ as catalyst and triethylamine as base in CH_2_Cl_2_ to give an isolated product in only 5% yield (Scheme 1). Further attempts to optimize the reaction conditions showed that the reaction mostly led to decomposition of the starting material in polar protic solvents or in the presence of bases or additives other than triethylamine (cf. Supporting Information File 1). However, the product was isolated in a moderate yield of 26% when the reaction was performed at room temperature in DMF as solvent. Notably, the yield was slightly lower (19%) when the reaction was performed at 40 °C, indicating that at higher temperatures side reactions are even more favored, such as the degradation of berberrubine under alkaline and/or oxidative conditions [35–37]. Most surprisingly, the NMR-spectroscopic data of the product were not consistent with the expected 9-*O*-arylated product. Moreover, in control experiments with 4-chlorophenylboronic acid (**3b**) the same unexpected product was formed. The product from the latter reaction was exemplarily studied by NOESY NMR experiments. Based on these results, the formation of the 10-*O*-arylated regioisomer **4b** was unambiguously confirmed by a clear NOE crosspeak between the protons 2’-H and 6’-H of the *O-*aryl group and the 11-H proton of the berberine (Figure S12, Supporting Information File 1), whereas an NOE between the *O-*aryl group and the 8-H proton, as expected for the 9-*O*-arylated product, was not observed. Furthermore, mass spectrometric data and elemental analysis data were consistent with the structure assignment of product **4b**.

**Scheme 2:**
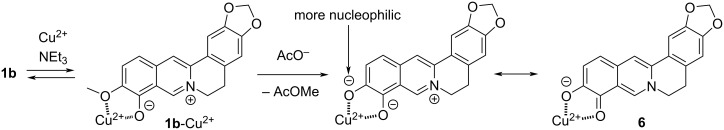
Cu^2+^-catalyzed demethylation of berberrubine (**1b**).

To assess whether the formation of the regioisomeric product **4a** is a general feature under these conditions, the reaction was conducted also with other representative boronic acids (Scheme 1). Although no products could be isolated after the reaction with 4-dimethylamino-, 3,4,5-trimethoxy- and 4-methylphenylboronic acid, a series of 10-*O*-arylated products **4b**–**f** was available in low yields (16–26%) by the Chan–Evans–Lam coupling of berberrubine (**1b**, Scheme 1). The formation of the 10-*O*-arylated products may be explained by an initial Lewis acid-catalyzed demethylation of berberrubine (**1b**) [38] to derivative **6** (Scheme 2) that reacts subsequently in the Cu^2+^-catalyzed coupling reaction with the boronic acid (Scheme 2). The regioselectivity of the latter reaction step is most likely determined by a stronger nucleophilicity of the oxyanion in the 10-position of **6** that is caused by the particular electron distribution in the intermediate **6**, specifically because of the decreased electron density at the 9-oxyanion by linear conjugation with the quaternary nitrogen atom [39]. Nevertheless, the reactivity of intermediate **6** is still relatively low, as indicated by the low yields of the Cu^2+^-catalyzed coupling reaction, thus resembling the parent berberrubine (**1b**), which has been shown to give no products in an Ullmann reaction [31]. Furthermore, the sensitivity of the isoquinolinium unit towards alkaline conditions and redox-active transition metal ions, especially under aerobic conditions, may also cause the low yields [35–37].

Unfortunately, the products **4a**–**f** are hardly soluble in aqueous solution, presumably due to their zwitterionic structure, which hampers their use in biological studies. To ensure a sufficient solubility, these derivatives were methylated in the 9-*O*-position by the reaction with iodomethane under mild alkaline conditions to give the corresponding 9-methoxy-substituted derivatives **5a**–**e** in moderate to good yields of 40–77% (Scheme 1). During the methylation of derivative **5f** a transesterification occurred and a 2:1 mixture of the ethyl and methyl ester was formed that could not be further separated. The novel compounds **4a**–**f** and **5a**–**e** were identified and fully characterized by NMR-spectroscopic analysis (^1^H, ^13^C, COSY, HSQC, HMBC), mass spectrometric data and elemental analyses.

### Absorption and emission properties

**Figure 2:**
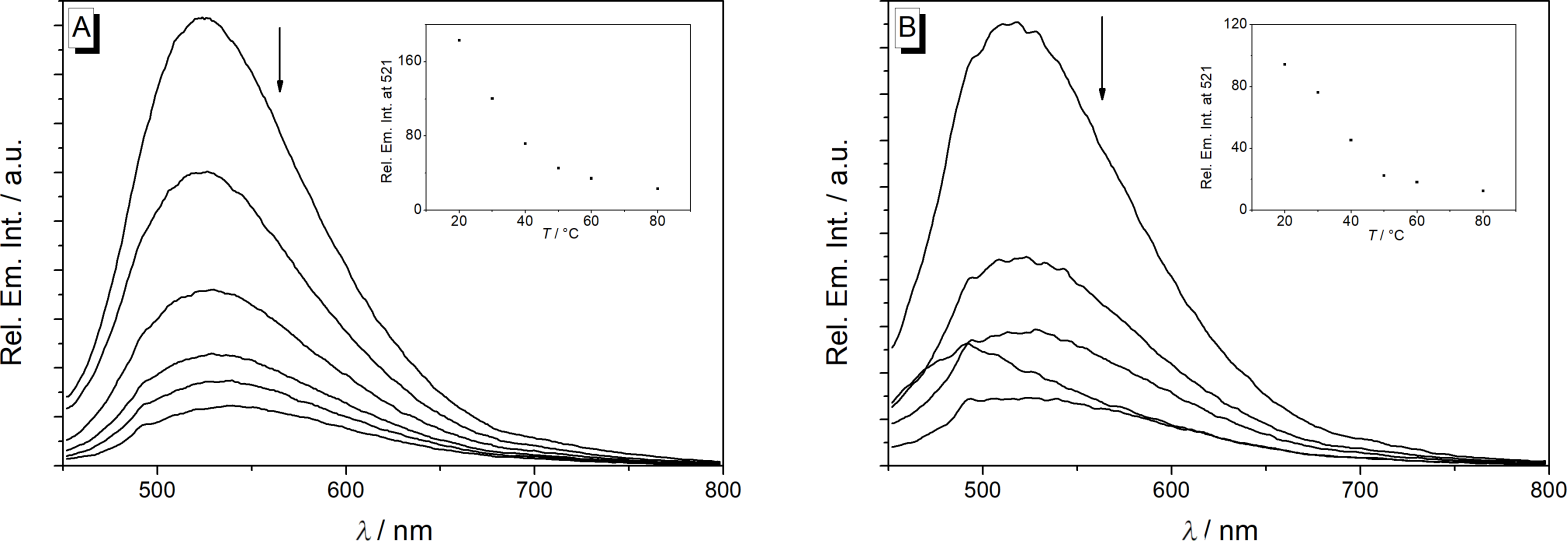
Temperature dependent emission spectra of derivatives **5a** and **5d** (*c* = 10 µM, with 0.25% v/v DMSO) in glycerol; λ_ex_ = 430 nm. The arrows indicate the changes of the emission intensity with increasing temperature. Insets: Plots of the relative fluorescence intensity versus the temperature of the solution.

The absorption properties of the derivatives **5a**–**e** resemble the ones of the berberine chromophore with long wavelength maxima between 403 nm (**5e** in aqueous buffer) and 426 nm (**5d** in CHCl_3_) (Table 1, Table S2, Figure S1, Supporting Information File 1) [40]. The emission intensity of all compounds is very low (Φ_fl_ << 0.01) and mostly not detectable in the series of tested solvents. To examine whether the low fluorescence quantum yields are caused by conformational changes in the excited state, the fluorescence was recorded in media with different viscosity, namely in glycerol at different temperatures (Figure 2, Figure S2, Supporting Information File 1). It was observed that the derivatives **5a**–**e** have significantly higher, but still relatively low emission quantum yields in glycerol at room temperature (Φ_fl_ = 0.009–0.031, 20 °C, η = 1412 cP) [41], whereas the emission intensity decreased with increasing temperature (Φ_fl_ = 0.001–0.002, 80 °C, η = 32 cP) [41]. Such a behavior usually indicates a radiationless deactivation of the excited state by conformational changes, e.g., torsional relaxation [42–43]. Since the fluorescence quantum yield of the parent berberine (**1a**) does not correlate well with the viscosity of the medium [40], it was concluded that the weak light-up effect in glycerol is mainly caused by the suppressed rotation about the Ar–O bond. Nevertheless, as the emission quantum yield of the derivatives **5a**–**e** still remained low, even at high viscosity of the medium, there obviously exist additional relaxation pathways in the excited state, most likely a photo-induced electron transfer (PET) from the 10-aryl substituent to the berberine chromophore. The latter has been shown to operate also in resembling cationic, biaryl-type dyes [42–43]. Along the same lines, the low intrinsic emission quantum yield of the parent berberine (**1a**) has been suggested to result from an internal charge transfer (ICT) process from the electron-rich benzodioxole unit to the isoquinolinium [44], which also contributes to the low fluorescence intensity of derivatives **5a**–**e**.

**Table 1:** Absorption and emission properties of representative 10-*O*-aryl-substituted berberine derivatives **5a** and **5d** in different solvents.

Compound	Solvent	λ_abs_^a^ [nm]	lg ε^b^	λ_fl_^c^ [nm]	Φ_fl_^d^

**5a**	CHCl_3_	420	3.85	521	0.02
	EtOH	418	3.86	545	<0.01
	MeOH	416	3.87	555	<0.01
	DMSO	412	3.82	^e^	^e^
	buffer	410	3.80	^e^	^e^
**5d**	CHCl_3_	426	3.85	525	0.02
	EtOH	422	3.86	541	<0.01
	MeOH	419	3.86	552	<0.01
	DMSO	415	3.84	^e^	^e^
	buffer	412	3.82	^e^	^e^

^a^Long-wavelength absorption maximum; *c*_L_ = 20 µM. ^b^ε = Molar extinction coefficient in cm^−1^ M^−1^. ^c^Fluorescence emission maximum; λ_ex_ = 415 nm. ^d^Fluorescence quantum yield relative to coumarin 153 in EtOH (Φ_fl_ = 0.544) [45]. ^e^Fluorescence quantum yield too low to be determined.

### DNA-binding properties

#### Thermal DNA denaturation experiments

For a first screening of the interactions of the derivatives **5a**–**e** with different G4-DNA forms, the effect of thermally induced unfolding of dye-labeled, quadruplex-forming oligonucleotides in the presence of the ligands was studied. In general, the binding of the ligand to the G4-DNA leads to a stabilization and thus to an increasing melting temperature of the DNA (Table 2). This effect of the ligands on the quadruplex melting temperature, *T*_m_, of the labeled G4-DNA **F21T** [fluo-G_3_(TTAG_3_)_3_-tamra; fluo = fluorescein, tamra = tetramethylrhodamine], **Fa2T** [fluo-(ACAG_3_TGT)_2_-tamra], **FmycT** [fluo-TGAG_3_TG_3_TAG_3_TG_3_TA-tamra] and **FkitT** [fluo-AG_3_AG_3_CGCTG_3_AG_2_AG_3_-tamra] was determined by fluorimetric monitoring of the temperature-dependent Förster resonance energy transfer (FRET) between the dyes [46]. The particular oligonucleotide sequences were chosen because they are known to be involved in biologically relevant processes, namely in the transcription regulation of myc (**FmycT**) [47–48], kit (**FkitT**) [49] and insulin (**a2**) [50], or in telomerase inhibition (**F21T**) [30,51–52]. At a ligand-DNA ratio (*LDR*) of 5, the derivatives **5b**–**e** induced a small, but significant shift of the melting temperature of the oligonucleotides **F21T** (∆*T*_m_ = 1.8–3.9 °C), and **FmycT** (∆*T*_m_ = 1.3–2.1 °C), while the melting temperatures of the sequences **Fa2T** and **FkitT** were only affected marginally by the presence of all ligands. Notably, derivative **5a** exhibited only a stabilizing effect on **F21T** (∆*T*_m_ = 3.2 °C), whereas the melting temperature of all other oligonucleotides was not affected significantly. The induced ∆*T*_m_ values of **F21T** are comparable to the one of the parent berberine (**1a**) under similar conditions (∆*T*_m_ = 3.1 °C) [27]. As compared to other known G4-DNA ligands [23,27–29], these shifts of the melting temperature in the presence of **5b**–**e** are rather small and indicate a relatively weak stabilizing effect on the quadruplex structures. Although the ∆*T*_m_ values do not necessarily correlate directly with the binding affinity [23], as they only refer to the stabilization at elevated temperatures, the data show that these ligands do not bind extremely strong to G4-DNA. Nevertheless, as these screening experiments revealed the most pronounced effect of the ligands on the ∆*T*_m_ values of **F21T**, the binding interactions with the corresponding unlabeled telomeric oligonucleotide sequence d[A(G_3_TTA)_3_G_3_] (**22AG**) were studied in more detail.

**Table 2:** Shift of melting temperature, ∆*T*_m,_ of oligonucleotides **F21T**, **Fa2T**, **FmycT** and **FkitT** in the presence of berberine derivatives **5a**–**e**.

	∆*T*_m_ [°C]^a^			
	
Ligand	**F21T**^b^	**Fa2T**^b^	**FkitT**^b^	**FmycT**^b^

**5a**	3.2	0.3	0.1	0.4
**5b**	2.6	1.2	0.4	1.3
**5c**	1.8	0.5	0.4	2.1
**5d**	3.9	0.1	0.6	1.6
**5e**	3.5	0.5	0.4	1.8

^a^Determined from fluorimetric analysis of dye-labeled oligonucleotides, *LDR* = 5; *c*_DNA_ = 0.2 mM (in oligonucleotides); KCl–LiCl–cacodylate buffer (*c*_K+_ = 10 mM, *c*_Na+_ = 10 mM, *c*_Li+_ = 90 mM, pH 7.0) λ_ex_ = 470 nm; λ_em_ = 515 nm; estimated error: ±0.5 °C. ^b^Dye-labeled oligonucleotides: **F21T** = fluo-G_3_(TTAG_3_)_3_-tamra, **Fa2T** = fluo-(ACAG_3_TGT)_2_-tamra, **FmycT** = fluo-TGAG_3_TG_3_TAG_3_TG_3_TA-tamra, **FkitT** = fluo-AG_3_AG_3_CGCTG_3_AG_2_AG_3_-tamra, fluo = fluorescein, tamra = tetramethylrhodamine.

#### Spectrometric titrations

**Figure 3:**
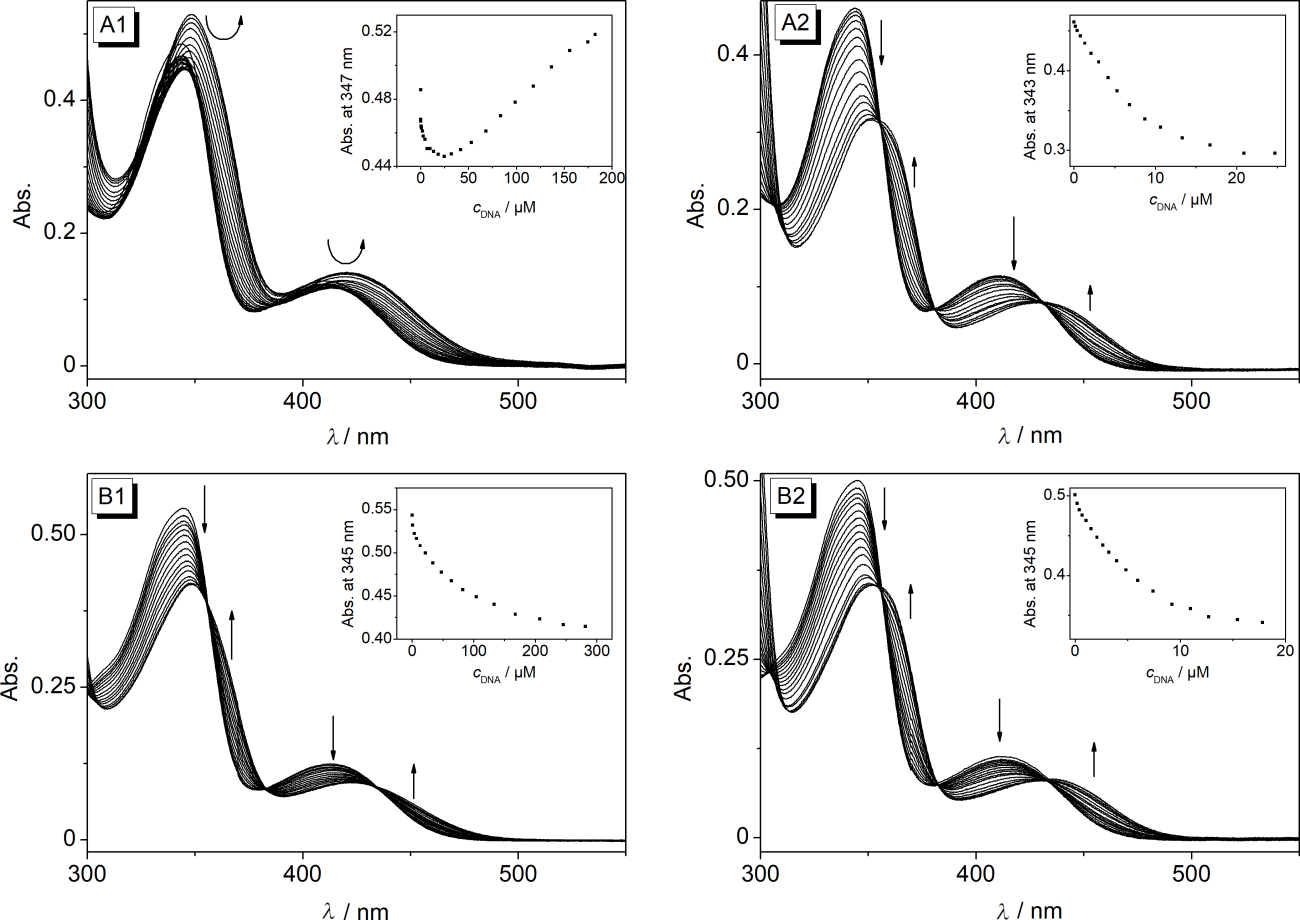
Photometric titration of **5a** (A) and **5d** (B) (*c*_Ligand_ = 20 μM) with ct DNA (1) in BPE buffer (*c*_Na+_ = 16 mM, pH 7.0, with 5% v/v DMSO) and with **22AG** (2) in K-phosphate buffer (*c*_K+_ = 110 mM, pH 7.0, with 5% v/v DMSO). The arrows indicate the changes of the absorption bands upon addition of DNA. Inset: Plot of the ligand absorption versus *c*_DNA_ (in base pairs).

The interactions of derivatives **5a**–**e** with calf thymus (ct) DNA, as a representative duplex DNA, and **22AG** were monitored by photometric and fluorimetric titrations. In almost all cases, a decrease of the absorption bands at 403–412 nm and 343 nm was observed upon addition of the DNA, along with a red shift of the absorption bands (Figure 3, Figure S3, Supporting Information File 1). Only the absorption of compound **5a** increased upon association with ct DNA. In general, a significant red shift of the absorption bands was observed during all titrations, which was more pronounced in the presence of **22AG** (∆λ = 18–24 nm) than in the presence of ct DNA (∆λ = 8–12 nm). However, no isosbestic points were observed during these titrations, which indicated different binding modes at the particular ligand-DNA ratios (*LDRs*). The resulting binding isotherms obtained from the photometric titrations were employed to determine the binding constants *K*_b_ (Table 3, Figure S5, Supporting Information File 1) [53]. As a general trend, all derivatives showed a slightly higher affinity towards G4-DNA (*K*_b_ = 5.2 × 10^5^ M^−1^ to 8.7 × 10^5^ M^−1^) than to ct DNA (*K*_b_ = 2.5 × 10^4^ M^−1^ to 5.1 × 10^4^ M^−1^). As compared with the parent berberine (**1a**), the binding affinity towards G4-DNA (**1a**: *K*_b_ = 4.5 × 10^5^ M^−1^) [16] is a bit higher, whereas the binding constants towards ct DNA are somewhat lower (**1a**: *K*_b_ = 9.7 × 10^4^ M^−1^) [54]. Hence the affinity of the ligands **5a**–**e** to duplex and quadruplex DNA lies in a similar range as the one of **1a**, so that the same DNA-targeted bioactivity of these substrates is assumed [14].

**Table 3:** Binding constants, *K*_b_, of **5a**–**e** with ct DNA and G4-DNA **22AG**, and fluorescence quantum yields of the DNA-bound ligands.

	ct DNA		**22AG**	
	
Ligand	*K*_b_ [10^4^ M^−1^]	Φ_fl_^a^	*K*_b_ [10^5^ M^−1^]	Φ_fl_^a^

**5a**	^b^	0.01	8.7 ± 0.2	0.02
**5b**	5.1 ± 0.6	0.01	7.1 ± 0.2	0.01
**5c**	4.0 ± 0.4	>0.01	5.2 ± 0.3	0.01
**5d**	2.5 ± 0.1	>0.01	7.2 ± 0.2	0.01
**5e**	4.1 ± 0.2	^c^	7.9 ± 0.3	^c^

^a^Fluorescence quantum yield relative to coumarin 153 in EtOH (Φ_fl_ = 0.544) [45]. ^b^Binding isotherms could not be fitted to a theoretical model. ^c^Fluorescence too low to be determined.

**Figure 4:**
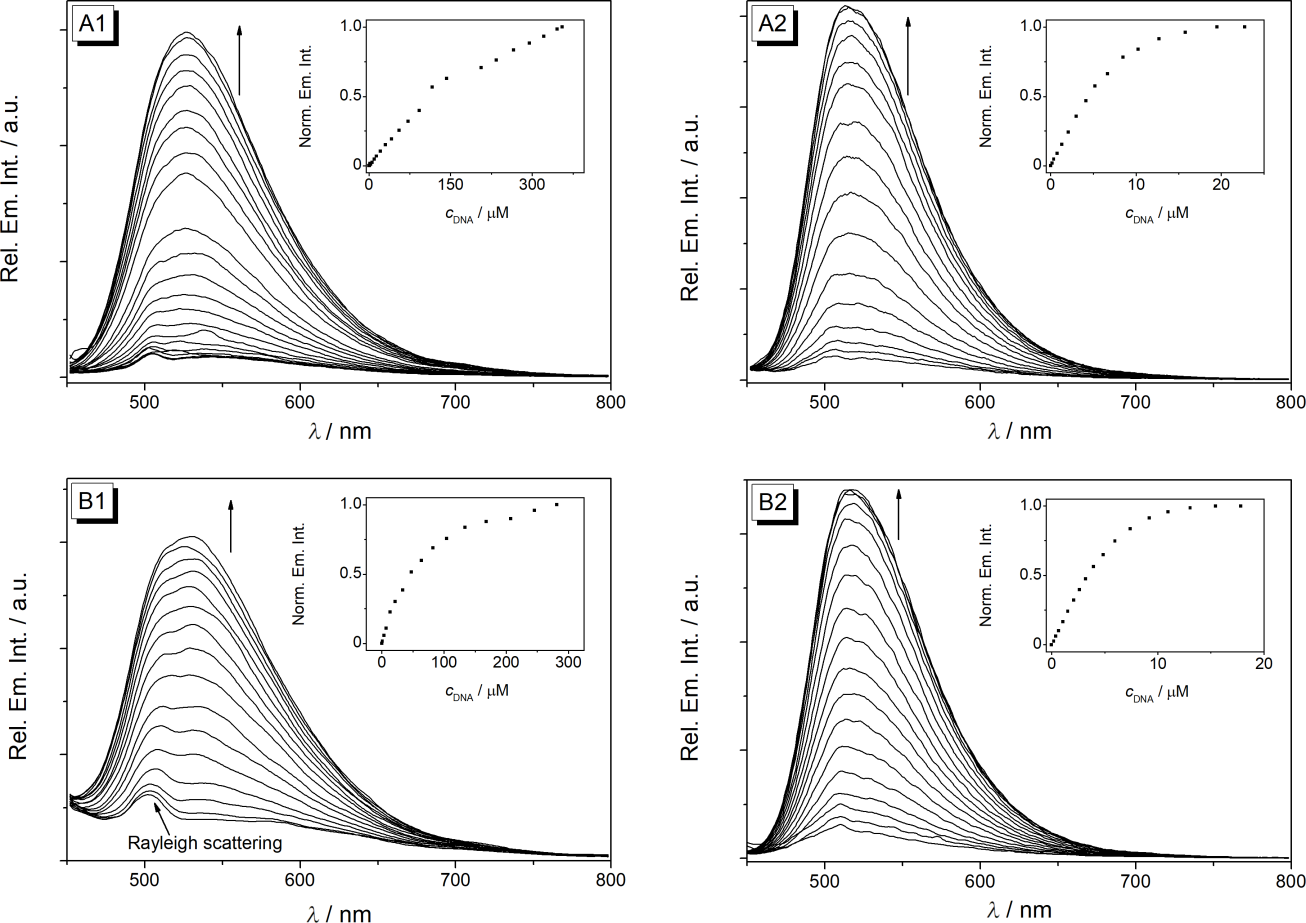
Fluorimetric titration of **5a** (A) and **5d** (B, *c*_Ligand_ = 20 μM) with ct DNA (1) in BPE buffer (*c*_Na+_ = 16 mM, pH 7.0, with 5% v/v DMSO) and with **22AG** (2) in K-phosphate buffer (*c*_K+_ = 110 mM, pH 7.0, with 5% v/v DMSO); λ_ex_ = 430 nm. The arrows indicate the changes of the emission bands upon addition of DNA. Insets: Plots of the relative fluorescence intensity versus *c*_DNA_ (in base pairs).

Upon addition of DNA the intensity of the emission bands of derivatives **5a**–**d**, that were hardly detectable in the absence of DNA, increased slightly with small shifts of the emission maxima (ct DNA: 520–529 nm; G4-DNA: 514–516 nm, Figure 4, Figure S4, Supporting Information File 1). Nevertheless, the fluorescence quantum yield remained rather low (Φ_fl_ < 0.01) for all DNA-bound derivatives with a slightly more pronounced increase in the presence of G4-DNA (Φ_fl_ = 0.010–0.017). In the case of derivative **5e**, the emission intensity remained essentially not detectable upon addition of DNA, presumably caused by the nitro group that is known to be an efficient emission quencher. It was demonstrated with experiments in glycerol solution that the emission of **5a**–**d** increases also upon suppression of conformational freedom of the molecule in media with high viscosity and limited free volume (Figure 2). Therefore, it is proposed that the association with the DNA causes a similar effect. Hence, the slightly increased emission intensity of the ligands **5a**–**d** upon addition of DNA supposedly originates from the restricted conformational freedom of the ligand within the DNA binding pocket, which suppresses the torsional relaxation as non-radiative deactivation pathway [42–43]. Thus, the relatively stronger emission enhancement upon binding of the ligands **5a**–**d** to G4-DNA is likely caused by a tighter accommodation of the aryl substituent within the G4-DNA binding pocket.

#### CD and LD spectroscopy

**Figure 5:**
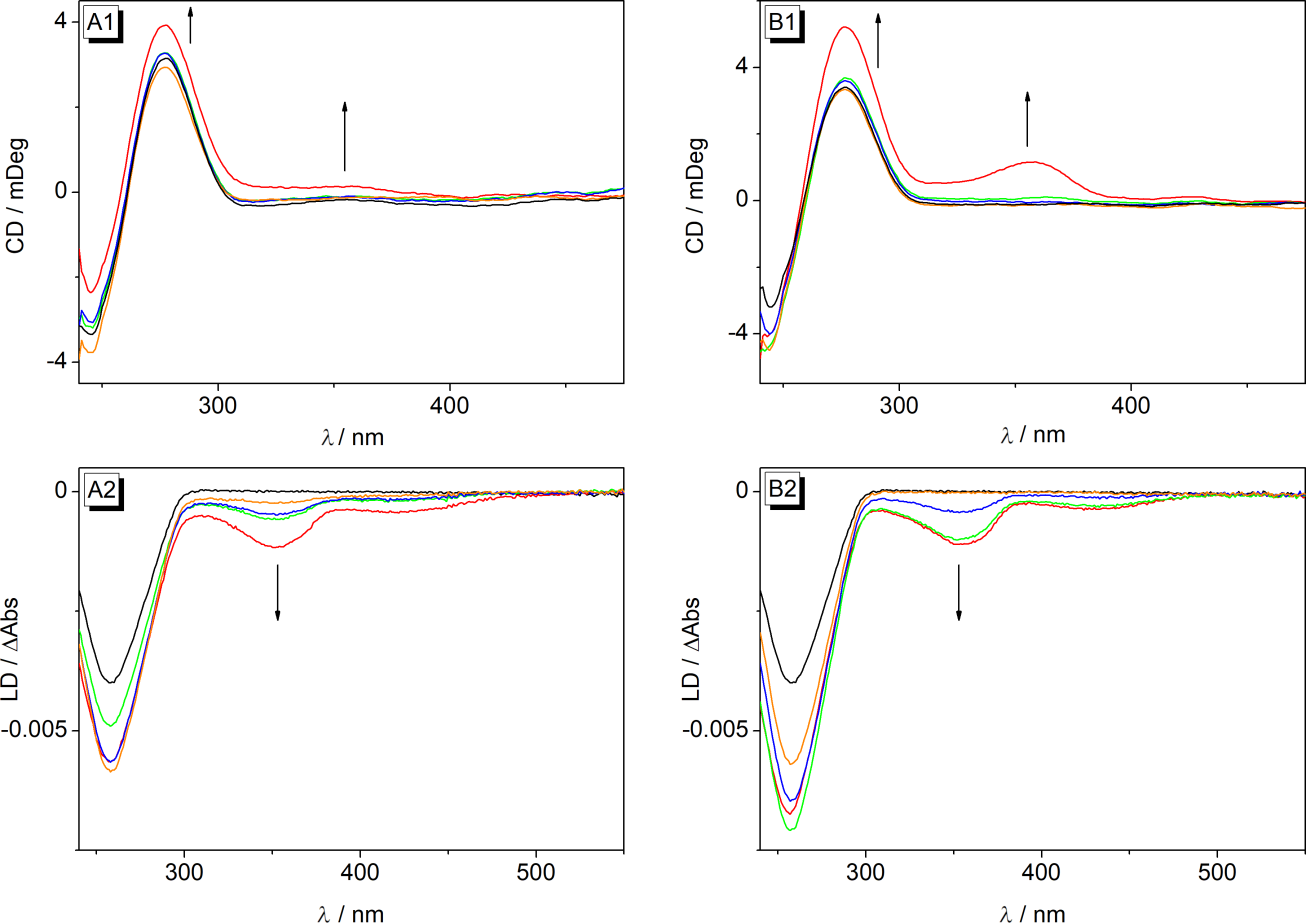
CD and LD spectra of ct DNA (1 and 2, *c*_DNA_ = 20 μM; in BPE buffer: 10 mM, pH 7.0; with 5% v/v DMSO) in the absence and presence of **5a** (A), and **5d** (B) at *LDR* = 0 (black), 0.05 (orange), 0.2 (blue), 0.5 (green), 1.0 (red). The arrows indicate the changes of CD and LD bands with increasing *LDR*.

Solutions of ligands **5a**–**e** in the presence of ct DNA were examined with flow linear dichroism (LD) and circular dichroism (CD) spectroscopy (Figure 5, Figure S6, Supporting Information File 1). The binding of ligands **5a**–**e** resulted in the development of negative LD signals at 350–355 nm and 420–429 nm clearly indicating an intercalative binding mode of the ligand, since these bands result from a coplanar alignment of the aromatic system of the ligand to the DNA bases [55–56]. Furthermore, all derivatives developed positive induced CD (ICD) signals in the absorption range of the ligands upon addition of ct DNA. However, except for ligand **5d** the intensity of the ICD signals remained rather low, which has already been observed for several berberine derivatives [25–26,57–58]. Altogether, especially considering the steric demand of the aryloxy substituent, the LD- and CD-spectroscopic data suggest a coplanar arrangement between DNA base pairs and the isoquinolinium unit pointing into the groove. This model is further supported by the relatively low fluorescence quantum yields of all ligands upon complexation to duplex DNA because in this structure the aryl substituent still has some conformational flexibility in the binding pocket leading to reduced fluorescence quantum yields.

The characteristic CD spectra of the G4-DNA **22AG** [57–58] only changed marginally in the presence of the derivatives **5a**–**e**; namely, only a small increase (**5b**) or a small decrease (**5a**, **5c–e**) of the characteristic CD band at 290 nm was observed with no obvious general trend. Most notably, a decrease of the shoulder at 255 nm was observed, in the case of **5e** together with a development of a new, slightly blue-shifted negative band, with increasing content of derivatives **5a**, **5b** and **5d**. This development of the CD spectrum is characteristic of a shift of the equilibrium between the [3 + 1] conformer, related to the shoulder at 255 nm [59], and the basket-type conformation of **22AG**, assigned to the positive signal at 290 nm and the weak negative band at 260 nm [60–61] in favor of the latter. Hence, these observations showed that the ligands bind preferentially to the basket-type quadruplex structure and thereby shift the equilibrium to this form. Furthermore, during all titrations of the derivatives **5a**–**e** to **22AG** no clear ICD band was detected, which is usually interpreted as an indication of terminal π stacking of the ligand to the quadruplex structure [62–64], however, this interpretation of an absent signal has to be applied very carefully.

## Conclusion

In summary, we demonstrated that 10-*O*-arylated berberine derivatives are accessible in low to moderate yield by the Chan–Evans–Lam coupling reaction of berberrubine (**1b**) and subsequent methylation. The straightforward synthetic route enables the synthesis of a new class of berberine derivatives from easily accessible starting materials. The derivatives bind with slightly higher affinity to G4-DNA as compared to the parent berberine (**1a**) and induce a moderate stabilization of telomeric quadruplex **22AG**. CD- and LD-spectroscopic studies revealed an intercalative binding mode with ct DNA and most likely terminal stacking as binding mode with G4-DNA. Lastly, all derivatives experienced a weak light-up effect upon complexation to DNA, which was slightly more pronounced upon binding to G4-DNA as compared to ct DNA. Since the ligands show essentially the same DNA binding properties as the parent berberine (**1a**), they have the potential to exhibit a similar DNA-targeted bioactivity. The latter may even be improved due to the higher lipophilicity and thus more balanced bioavailability of the ligands [65]. Accordingly, the activity of these derivatives in the treatment of tuberculosis [7], diarrhea [8], diabetes [9], cardiovascular diseases [10] or high cholesterol levels [11] is worth to be tested, because the parent berberine is already employed as drug for these diseases. In summary, a new complementary class of arylated berberine derivatives was discovered, which may constitute a promising starting point for the development of lead structures in drug discovery.

## Experimental

### Equipment

Absorption spectra: Varian Cary 100 Bio spectrophotometer in quartz cells (10 mm × 4 mm) with baseline correction. Emission spectra: Varian Cary Eclipse spectrophotometer in quartz cells (10 mm × 4 mm) at 20 °C. NMR spectra: Jeol ECZ 500 (^1^H: 500 MHz, ^13^C: 125 MHz) at 25 °C; processed with MestReNova software and referenced to the solvent [δ(DMSO-*d*_5_): ^1^H = 2.50 ppm, ^13^C: δ = 39.5 ppm, δ(CHCl_3_): ^1^H = 7.26 ppm, ^13^C: δ = 77.2 ppm). Elemental analyses: HEKAtech EUROEA combustion analyzer, determined by Rochus Breuer, Organische Chemie I, Universität Siegen. Mass spectra (ESI): Finnigan LCQ Deca (*U* = 6 kV; working gas: Ar; auxiliary gas: N_2_; temperature of the capillary: 200 °C). Circular dichroism (CD) and linear dichroism (LD): Chirascan CD spectrometer, Applied Photophysics. Flow-LD: High Shear Cuvette Cell Accessory (Applied Photophysics); the LD samples were recorded in a rotating cuvette with a shear gradient of 1200 s^−1^. Melting points: Büchi 545 (Büchi, Flawil, CH).

### Materials

Berberrubine (**1b**) was synthesized according to the published protocol [66]. Calf thymus DNA (ct DNA, type I; highly polymerized sodium salt; ε = 12824 cm^−1^ M^−1^) [67] was purchased from Sigma-Aldrich (St. Louis, USA) and used without further purification. Concentration of ct DNA is given in base pairs (bp). The ct DNA was dissolved in BPE buffer solution. Oligodeoxyribonucleotides (HPLC purified) d[A(GGGTAA)_3_GGG] (**22AG**), **F21T** [fluo-G_3_(TTAG_3_)_3_-tamra; fluo = fluorescein, tamra = tetramethylrhodamine], **Fa2T** [fluo-(ACAG_3_TGT)_2_-tamra], **FmycT** [fluo-TGAG_3_TG_3_TAG_3_TG_3_TA-tamra] and **FkitT** [fluo-AG_3_AG_3_CGCTG_3_AG_2_AG_3_-tamra] were purchased from Biomers.net GmbH (Ulm, Germany). Oligonucleotides were dissolved in K-phosphate buffer, and the solution was heated to 95 °C for 5 min and then cooled slowly to room temperature within 4 h. K-phosphate buffer: 25 mM K_2_HPO_4_, 60 mM KCl; adjusted with 50 mM KH_2_PO_4_, 60 mM KCl to pH 7.0; BPE (biphosphate EDTA) buffer: 6.0 mM Na_2_HPO_4_, 2.0 mM NaH_2_PO_4_, 1.0 mM Na_2_EDTA; pH 7.0. All buffer solutions were prepared from purified water (resistivity 18 MΩ cm) and biochemistry-grade chemicals. The buffer solutions were filtered through a PVDF membrane filter (pore size 0.45 μm) prior to use.

## Supporting Information

File 1 Experimental procedures, syntheses, additional spectroscopic data, ^1^H NMR and ^13^C NMR spectra.
