# Supplementary material for: Synthesis of 10-O-aryl-substituted berberine derivatives by Chan–Evans–Lam coupling and investigation of their DNA-binding properties
Source: Beilstein J Org Chem. 2021 May 4;17:991–1000. doi: 10.3762/bjoc.17.81 (PMC8111429; doi:10.3762/bjoc.17.81)
Supplement: File 1 — Experimental procedures, syntheses, additional spectroscopic data, 1H NMR and 13C NMR spectra. [file Beilstein_J_Org_Chem-17-991-s001.pdf]

BEILSTEIN JOURNAL OF ORGANIC CHEMISTRY

Supporting Information

for

Synthesis of 10-*O*-aryl-substituted berberine derivatives by Chan–Evans–Lam coupling and investigation of their DNA-binding properties

Peter Jonas Wickhorst, Mathilda Blachnik, Denisa Lagumdzija and Heiko Ihmels

Beilstein J. Org. Chem. **2021,** *17,* 991–1000. doi:10.3762/bjoc.17.81

Experimental procedures, syntheses, additional spectroscopic data, ^{1}H NMR and ^{13}C NMR spectra

License and Terms: This is a supporting information file under the terms of the Creative Commons Attribution License (https://creativecommons.org/licenses/by/4.0). Please note that the reuse, redistribution and reproduction in particular requires that the author(s) and source are credited and that individual graphics may be subject to special legal provisions.
The license is subject to the *Beilstein Journal of Organic Chemistry* terms and conditions: (https://www.beilstein-journals.org/bjoc/terms)

Table of Contents

1. Synthesis	S2
2. Photophysical properties	S10
3. Determination of the fluorescence quantum yields	S12
4. Photometric and fluorimetric DNA titrations	S13
5. CD- and LD-spectroscopic analysis	S17
6. Thermal DNA-denaturation experiments	S18
7. NMR spectra	S20
8. References	S32

1. Synthesis

Generall Procedure for the Chan–Evans–Lam coupling reaction of berberrubine with arylboronic acids (GP 1).

A mixture of berberrubine (**1b**, 240 mg, 750 µmol), $Cu(OAc)_2$ (150 mg, 750 µmol), triethylamine (420 µL, 3.00 mmol), molecular sieves (4 Å) and the respective arylboronic acid (3.00 mmol) in DMF (5 mL) was stirred for 16 h at rt. To the reaction mixture was added CH_2Cl_2 (100 mL) and aq. HBr solution (1 M, 30 mL), and the organic layer was separated. The aqueous layer was extracted with CH_2Cl_2 (30 mL), and the combined organic layers were washed with saturated aqueous $NaHCO_3$ solution (20 mL), separated, and dried with Na_2SO_4. The drying agent was filtered off and the solvent was removed by destillation. The crude product was purified by flash column chromatography (SiO_2, CH_2Cl_2/MeOH 0% → 7%) and washed with acetone (20 mL) to obtain the desired product.

10-Phenoxy-5,6-dihydro-[1,3]dioxolo[4,5-g]isoquinolino[3,2-a]isoquinolin-7-ium-9-olate (**4a**).

According to GP 1 the reaction of **1b** (240 mg, 750 µmol) with **3a** (365 mg, 3.00 mmol) gave product **4a** as red solid (74.8 mg, 195 µmol, 26%); mp > 300 °C (dec.). – ^{1}H NMR of **4a** x CF_3COOD (500 MHz, DMSO-d_6 + CF_3COOD): δ = 3.21 (t, ^3J = 6 Hz, 2H, 5-H), 4.93 (t, ^3J = 6 Hz, 2H, 6-H), 6.18 (s, 2H, OCH_2O), 7.42 (d, ^3J = 4 Hz, 4‘-H), 7.09 (s, 1H, 4-H), 7.18 (t, ^3J = 9 Hz, 2H, 3‘-H, 5‘-H), 7.03 (t, ^3J = 9 Hz, 2H, 2‘-H, 6‘-H), 7.66 (d, ^3J = 4 Hz, 1H, 12-H), 7.80 (s, 1H, 1-H), 7.82 (s, 1H, 11-H), 8.91 (s, 1H, 13-H), 10.00 (s, 1 H, 8-H). – ^{13}C NMR of **4a** x CF_3COOD (125 MHz, DMSO-d_6 + CF_3COOD): δ = 26.3 (C5), 54.9 (C6), 102.1 (OCH_2O), 105.6 (C1), 108.4 (C4), 117.4 (C4’), 118.7 (C12), 119.1 (C13b), 119.9 (C8a), 120.4 (C13), 123.5 (C3’, C5’), 130.1 (C2’, C6’), 131.0 (C4a), 132.1 (C11), 135.8 (C12a), 138.6 (C13a), 140.6 (C9), 145.9 (C8), 146.8 (C10), 147.7 (C2), 150.0 (C3), 156.9 (C1’). – MS (ESI$^+$): *m/z* (%) = 384 (100) $[M+H]^+$. – El. Anal. for $C_{24}H_{17}NO_4$, calcd. (%): C 75.19, H 4.47, N 3.65, found (%): C 74.86, H 4.22, N 3.67.

10-(4-Chlorophenoxy)-5,6-dihydro-[1,3]dioxolo[4,5-g]isoquinolino[3,2-a]isoquinolin-7-ium-9-olate (**4b**).

According to GP 1 the reaction of **1b** (240 mg, 750 µmol) with **3b** (469 mg, 3.00 mmol) gave product **4b** as red solid (53.0 mg, 127 µmol, 17%); mp > 286 °C (dec.). – ^{1}H NMR of **4b** (500 MHz, $CDCl_3$): δ = 3.15 (t, ^{3}J = 7 Hz, 1H, 5-H), 4.59 (t, ^{3}J = 7 Hz, 1H, 6-H), 6.09 (s, 1H, OCH_2O), 6.72 (s, ^{3}J = 7 Hz, 1H, 12-H), 6.81 (s, 1H, 4-H), 6.92 (d, ^{3}J = 7, 2H, 2’-H, 6’-H), 7.12 (d, ^{3}J = 7 Hz, 2H, 3’-H, 5’-H), 7.33 (s, 1H, 1-H), 7.57 (d, ^{3}J = 7 Hz, 1H, 11-H), 7.86 (s, 1H, 13-H), 9.30 (s, 1 H, H8). – ^{13}C NMR of **4b** (125 MHz, $CDCl_3$): δ = 28.2 (C5), 54.2 (C6), 102.2 (OCH_2O), 105.0 (C1), 108.6 (C4), 117.9 (C2‘, C6‘), 118.5 (C13), 121.4 (C13b), 126.6 (C4‘) 129.2 (C4a), 129.3 (C3‘, C5‘) 133.1 (C11), 135.9 (C13a), 136.5 (C12a), 144.7 (C10), 146.2 (C8), 148.4 (C2), 150.6 (C3), 157.3 (C1‘), 165.8 (C9). – MS (ESI^{+}): *m/z* (%) = 419 (100) $[M+H]^{+}$ – El. Anal. for $C_{24}H_{16}NO_4Cl$ x 2/3 H_2O, calcd. (%): C: 67.06; H: 4.06; N: 3.26; found (%): C: 66.76, H: 3.50, N: 3.26.

10-(4-Bromophenoxy)-5,6-dihydro-[1,3]dioxolo[4,5-g]isoquinolino[3,2-a]isoquinolin-7-ium-9-olate (**4c**).

According to GP 1 the reaction of **1b** (240 mg, 750 µmol) with **3c** (602 mg, 3.00 mmol) gave product **4c** as red solid (65.0 mg, 141 µmol, 19%); mp > 300 °C (dec.). – ^{1}H NMR of **4c** x CF_3COOD (500 MHz, DMSO-d_6 + CF_3COOD): δ = 3.21 (t, ^{3}J = 6 Hz, 2H, 5-H), 4.93 (t, ^{3}J = 6 Hz, 2H, 6-H), 6.18 (s, 2H, OCH_2O), 6.98 (d, ^{3}J = 9 Hz, 2H, 2‘-H, 6‘-H), 7.09 (s, 1H, 4-H), 7.58 (d, ^{3}J = 9 Hz, 2H, 3‘-H, 5‘-H), 7.67 (d, ^{3}J = 9 Hz, 1H, 12-H), 7.82 (s, 1H, 1-H), 7.86 (d, ^{3}J = 9 Hz, 1H, 11-H), 8.91 (s, 1H, 13-H), 9.99 (s, 1H, 9-H). – ^{13}C NMR of **4c** x CF_3COOD (100 MHz, DMSO-d_6 + CF_3COOD): δ = 26.3 (C5), 54.9 (C6), 102.1 (OCH_2O), 105.6 (C1), 108.4 (C4), 115.0 (C4’), 118.8 (C12), 119.2 (C8a), 119.4 (C2‘, C6‘), 120.0 (C13), 120.3 (C13b), 131.0 (C4a), 132.3 (C11), 132.7 (C3‘, C5‘), 136.1 (C13a), 138.8 (C12a), 140.0 (C9), 146.0 (C8), 147.0 (C10), 147.7 (C2), 150.1 (C3), 156.4 (C1‘). – MS (ESI^{+}): *m/z* (%) = 462 (100) $[M+H]^{+}$. – El. Anal. for $C_{24}H_{16}BrNO_4$, calcd. (%): C 62.35, H 3.49, N 3.03, found. (%): C 61.92, H 3.23, N 3.07.

10-(4-Methoxyphenoxy)-5,6-tetradro-[1,3]dioxolo[4,5-g]isoquinolino[3,2-a]isoquinolin-7-ium-9-olate (**4d**).

According to GP 1 the reaction of **1b** (240 mg, 750 µmol) with **3d** (455 mg, 3.00 mmol) gave product **4d** as red solid (54.5 mg, 132 µmol, 18%); mp > 300 °C (dec.). – ^{1}H NMR of **4d** x CF_3COOD (500 MHz, DMSO-d_6 + CF_3COOD): δ = 3.21 (t, ^{3}J = 6 Hz, 2H, 5-H), 3.76 (s, 3H, 4´-OCH3), 4.93 (t, ^{3}J = 6 Hz, 2H, 6-H), 6.17 (s, 2H, OCH_2O), 6.99 (d, ^{3}J = 9 Hz, 2H, 2‘-H, 6‘-H), 7.04 (d, ^{3}J = 9 Hz, 2H, 3‘-H, 5‘-H), 7.09 (s, 1H, 4-H), 7.64 (d, ^{3}J = 4 Hz, 1H, 12-H), 7.72 (d, ^{3}J = 4 Hz, 1H, 11-H), 7.82 (s, 1H, 1-H), 8.88 (s, 1H, 13-H), 9.99 (s, 1H, 8-H). – ^{13}C NMR of **4d** x CF_3COOD (125 MHz, DMSO-d_6 + CF_3COOD): δ= 26.3 (C5), 54.9 (C6), 55.5 (OCH_3), 102.1 (OCH_2O), 105.5 (C1), 108.4 (C4), 115.1 (C2‘, C6‘), 118.5 (C12), 118.9 (C3‘, C5‘), 119.5 (C13), 119.9 (C13b), 120.4 (C8a), 130.8 (C4a), 130.9 (C11), 135.1 (C12a), 138.2 (C13a), 142.1 (C9), 145.8 (C8), 145.9 (C10), 149.9 (C4‘), 155.7 (C1‘). – MS (ESI^{+}): *m/z* (%) = 414 (100) $[M+H]^{+}$. – El. Anal. for $C_{25}H_{20}NO_5$ x $1.5H_2O$, calcd. (%): C 68.17, H 5.03, N 3.18, found (%): C 68.44, H 4.57, N 3.31.

10-(4-Nitrophenoxy)-5,6-dihydro-[1,3]dioxolo[4,5-g]isoquinolino[3,2-a]isoquinolin-7-ium-9-olate (**4e**).

According to GP 1 the reaction of **1b** (150 mg, 467 µmol) with **3e** (234 mg, 1.40 mmol) gave product **4e** as red solid (43.4 mg, 101 µmol, 22%); mp > 300 °C (dec.). – ^{1}H NMR of **4e** x CF_3COOD (500 MHz, DMSO-d_6 + CF_3COOD): δ = 3.22 (t, ^{3}J = 6 Hz, 2H, 5-H), 4.94 (t, ^{3}J = 6 Hz, 2H, 6-H), 6.18 (s, 2H, OCH_2O), 7.10 (s, 1H, 4-H), 7.15–7.17 (d, ^{3}J = 9 Hz, 2H, 2‘-H, 6‘-H), 7.72 (d, ^{3}J = 9 Hz, 1H, 12-H), 7.84 (s, 1H, 1-H), 7.97–7.99 (m, 1H, 11-H), 8.28–8.30 (m, 2H, 3´-H, 5´-H), 8.95 (s, 1H, 13-H), 10.03 (s, 1H, 8-H). – ^{13}C NMR of **4e** x CF_3COOD (125 MHz, DMSO-d_6 + CF_3COOD): δ = 26.4 (C5), 54.9 (C6), 102.2 (OCH_2O), 105.7 (C1), 108.5 (C4), 2 x 116.9 (C2’, C6’), 118.9 (C12), 119.5 (C8a), 120.0 (C13), 120.3 (C13b), 126.1 (C3’, C5’), 131.2 (C4a), 133.2 (C11), 137.0 (C9), 138.5 (C12a), 139.3 (13aC), 142.6 (C1’), 147.8 (C2), 150.2 (C3), 158.1 (C10), 162.2 (C4’), 146.2 (C8). – MS (ESI^{+}): *m/z* (%) = 464 (100) $[M+2H_2O]^{+}$. – El. Anal. for $C_{25}H_{20}NO_5$ x $1.5H_2O$, calcd. (%): C 63.72, H 4.16, N 6.19, found (%): C 63.39, H 3.57, N 6.01.

10-(4-(Ethoxycarbonyl)phenoxy)-5,6-dihydro-[1,3]dioxolo[4,5-g]isoquinolino[3,2-a]isoquinolin-7-ium-9-olate (**4f**).

According to GP 1 the reaction of **1b** (240 mg, 750 µmol) with **3f** (579 mg, 3.00 mmol) gave product **4f** as red solid (54.1 mg, 119 µmol, 16%); mp > 278° C (dec.). – ^{1}H NMR of **4f** x CF_3COOD (500 MHz, DMSO-d_6 + CF_3COOD): δ = 1.31 (q, ^3J = 6 Hz, 3H C**H**$_3$CH$_2$O), 3.21 (t, ^3J = 6 Hz, 2H, 5-H), 4.31 (t, ^3J = 6 Hz, 2H CH$_3$C**H**$_2$O), 4.93 (t, ^3J = 6 Hz, 2H, 6-H), 6.18 (s, 1 H, OCH$_2$O), 7.09 (s, 1H, 4-H), 7.09 (t, ^3J = 6 Hz, 2H, 2‘-H, 6‘-H), 7.69 (t, ^3J = 6 Hz, 1H, 12-H), 7.83 (s, 1H, 1-H), 7.91 (t, ^3J = 6 Hz, 11-H), 8.00 (t, ^3J = 6 Hz, 3‘-H, 5‘-H), 8.93 (s, 1H, 13-H), 10.01 (s, 1H, 8-H). – ^{13}C NMR of **4f** x CF_3COOD (125 MHz, DMSO-d_6 + CF_3COOD): δ = 14.19 (**C**H$_3$CH$_2$O), 26.3 (C5), 54.9 (C6), 60.65 (CH$_3$**C**H$_2$O), 102.2 (OCH$_2$O), 105.7 (C1), 108.5 (C4), 116.7 (C2’, C6’), 118.9 (C12), 119.4 (C8a), 120.0 (C13), 120.3 (C13b), 124.6 (C4’), 131.1 (C4a), 133.0 (C11), 134.5 (C3’, C5’), 136.6 (C9), 139.1 (C13a), 139.1 (C12a), 146.0 (C8), 147.4 (C10), 147.5 (C2), 150.1 (C3), 165.1 (C=O), 165.1 (C1’). – MS (ESI$^+$): *m/z* (%) = 454 (100) [M]$^+$. – El. Anal. for $C_{27}H_{21}NO_6$ x 0.5 H_2O, calcd. (%): C 69.82, H 4.77, N 3.02, found (%): C 69.39, H 4.10, N 2.98.

General Procedure for the *O*-methylation of derivatives 5a–e (GP 2).

A mixture of the respective aryloxyisoquinolinium (36.2–67.5 µmol) and K_2CO_3 (1 molar eq.), and MeI (1.5 ml, 3.42 g, 24.1 mmol) in CH_2Cl_2 (3 ml) was stirred for 2 d at r.t. The mixture was diluted with CH_2Cl_2 (30 ml) and filtered, and the solvent was removed by distillation. The residue was dissolved in MeOH (5 ml) and diluted with water (5 ml). A solution of aq. $NaBF_4$ (sat., 1.0 ml) was added. The yellow precipitate was filtered off and redissolved in CH_2Cl_2 (30 ml). The suspension was filtered and the solvent was removed to give the essentially pure product. Analytical pure samples were obtained by recrystallization from MeOH/EtOAc.

9-Methoxy-10-phenoxy-5,6-dihydro-[1,3]dioxolo[4,5-g]isoquinolino[3,2-a]isoquinolin-7-ium tetrafluoroborate (**5a**).

According to GP 2 the reaction of **4a** (28.1 mg, 73.5 µmol), with MeI (1.5 ml, 3.42 g, 24.1 mmol) and K_2CO_3 (10.2 mg, 73.5 µmol) gave product **5a** as yellow solid (27.2 mg, 56.2 µmol, 76%); mp > 288 °C (dec.). –^{1}H NMR (500 MHz, DMSO-d_6): δ = 3.23 (t, ^3J= 6 Hz, 2 H, 5-H), 4.15 (s, 3H, OCH_3), 4.96 (t, ^3J = 6 Hz, 2H, 6-H), 6.19 (s, 2H, OCH_2O), 7.42 (d, ^3J = 4 Hz, 1H, 4'-H), 7.09 (d, ^3J = 8 Hz, 2H, 2'-H, 6'-H), 7.11 (s, 1H, 4-H), 7.21 (t, ^3J = 8 Hz, 1H, 4'-H), 7.45 (d, ^3J = 8 Hz, 2H, 3'-H, 5'-H), 7.84 (d, ^3J = 4 Hz, 1H, 1-H), 7.90 (d, ^3J = 9 Hz, 1H, 11-H), 7.95 (s, ^3J = 9 Hz, 1H, 12-H), 9.00 (s, 1H, 13-H), 9.98 (s, 1H, 8-H). – ^{13}C NMR (125 MHz, DMSO-d_6): δ = 26.3 (C5), 55.2 (C6), 62.3 (CH_3), 102.2 (OCH_2O), 105.7 (C1), 108.5 (C4), 117.4 (C2', C6'), 120.2 (C13b), 120.3 (C13), 121.9 (C8a), 123.6 (C12), 123.9 (C4'), 130.3 (C3', C5'), 131.2 (C4a), 132.9 (C11), 136.1 (C12a), 139.3 (C13a), 145.1 (C10), 145.8 (C8), 147.2 (C9), 147.8 (C2), 150.2 (C3), 156.5 (C1'). – MS (ESI$^+$): *m/z* (%) = 398 (100) [M-BF_4]$^+$. – El. Anal. for $C_{25}H_{20}NO_4BF_4$, calcd. (%): C 61.88, H 4.15, N 2.89, found (%): C 61.98, H 3.86, N 2.82.

9-Methoxy-10-(4-chlorophenoxy)-5,6-dihydro-[1,3]dioxolo[4,5-g]isoquinolino[3,2-a]isoquinolin-7-ium tetrafluoroborate (**5b**).

According to GP 2 the reaction of **4b** (28.2 mg, 67.5 µmol), with MeI (1.5 ml, 3.42 g, 24.1 mmol) and K_2CO_3 (9.33 mg, 67.5 mmol) gave product **5b** as yellow solid (22.4 mg, 43.1 µmol, 64%); mp > 268 °C (dec.). –^{1}H NMR (500 MHz, DMSO-d_6): δ = 3.21 (t, ^3J = 6 Hz, 2H, 5-H), 4.13 (s, 3H, CH_3), 4.96 (t, ^3J = 6 Hz, 2H, 6-H), 6.19 (s, 2H, OCH_2O), 7.11 (s, 1H, 4-H), 7.13 (d, ^3J = 9 Hz, 2H, 2'-H, 6'-H), 7.50 (d, ^3J = 9 Hz, 2H, 3´-H, 5´-H), 7.85 (s, 1H, 1-H), 7.94 (d, ^3J = 9 Hz, 1H, 11-H), 7.96 (d, ^3J = 9 Hz, 1H, 12-H), 9.01 (s, 1H, 13-H), 9.98 (s, 1H, 8-H). – ^{13}C NMR (125 MHz, DMSO-d_6): δ = 26.3 (C5), 55.2 (C6), 62.4 (CH_3), 102.2 (OCH_2O), 105.7 (C1), 108.5 (C4), 119.1 (C2', C6'), 120.2 (C13b), 120.3 (C13), 121.9 (C8a), 123.8 (C12), 127.8 (C4'), 130.1 (C3', C5'), 131.2 (C4a), 132.9 (C11), 136.4 (C12a), 139.5 (C13a), 144.8 (C10), 145.9 (C8), 147.4 (C9), 147.8 (C2), 150.3 (C3), 155.4

(C1’). – MS (ESI$^+$): *m/z* (%) = 432 (100) [M-BF_4]$^+$. – El. Anal. for $C_{25}H_{19}NO_4BClF_4$, calcd. (%): C 57.78, H 3.69, N 2.70, found (%): C 57.84, H 3.37, N 2.72.

9-Methoxy-10-(4-bromophenoxy)-5,6-dihydro-[1,3]dioxolo[4,5-g]isoquinolino[3,2-a]isoquinolin-7-ium tetrafluoroborate (**5c**).

According to GP 2 the reaction of **4c** (19.2 mg, 41.6 µmol) with MeI (1.5 ml, 3.42 g, 24.1 mmol) and K_2CO_3 (5.75 mg, 41.6 µmol) gave product **5c** as yellow solid (17.6 mg, 31.2 µmol, 75%); mp > 264 °C (dec.). – ^{1}H NMR (500 MHz, DMSO-d_6): δ = 3.22 (t, ^3J = 6 Hz, 2H, 5-H), 4.13 (s, 3H, CH_3), 4.96 (t, ^3J = 6 Hz, 2H, 6-H), 6.19 (s, 2H, OCH_2O), 7.06 (d, ^3J = 9 Hz, 2H, 2‘-H, 6‘-H), 7.11 (s, 1H, 4-H), 7.62 (d, ^3J = 9 Hz, 2H, 3´-H, 5´-H), 7.83 (s, 1H, 1-H), 7.94 (d, ^3J = 9 Hz, 1H, 12-H), 7.96 (d, ^3J = 9 Hz, 1H, 11-H), 9.01 (s, 1H, 13-H), 9.98 (s, 1H, 8-H). – ^{13}C NMR (125 MHz, DMSO-d_6): δ = 26.3 (C5), 55.2 (C6), 62.4 (CH_3), 102.2 (OCH_2O), 105.7 (C1), 108.5 (C4), 115.7 (C4‘) 119.5 (C2’, C6’), 120.2 (C13b), 120.3 (C13), 121.9 (C8a), 123.7 (C12), 131.2 (C4a), 133.0 (C11), 133.0 (C3’, C5’), 136.4 (C12a), 139.5 (C13a), 144.6 (C10), 145.9 (C8), 147.4 (C9), 147.8 (C2), 150.3 (C3), 155.9 (C1’). – MS (ESI$^+$): *m/z* (%) = 476 (100) [M-BF_4]$^+$. – El. Anal. for $C_{25}H_{19}BrNO_4BF_4$, calcd. (%): C 52.39, H 3.52, N 2.44, found. (%): C 52.33, H 2.99, N 2.31.

9-Methoxy-10-(4-Methoxyphenoxy)-5,6-dihydro-[1,3]dioxolo[4,5-g]isoquinolino[3,2-a]isoquinolin-7-ium tetrafluoroborate (**5d**).

According to GP 2 the reaction of **4d** (15.0 mg, 36.2 µmol) with MeI (1.5 ml, 3.42 g, 24.1 mmol) and K_2CO_3 (5.00 mg, 36.2 µmol) gave product **5d** as yellow solid (7.45 mg, 14.5 µmol, 40%); mp > 260 °C (dec.). – ^{1}H NMR (500 MHz, DMSO-d_6): δ = 3.22 (t, ^3J = 6 Hz, 2H, 5-H), 3.77 (s, 3H, 4-OCH_3), 4.17 (s, 3H, 9-OCH_3), 4.95 (t, ^3J = 6 Hz, 2H, 6-H), 6.18 (s, 2H, OCH_2O), 7.01–7.03 (m, 2H, 2‘-H, 6‘-H), 7.08–7.10 (m, 2H, 3´-H, 5´-H), 7.11 (m, 1H, 4-H), 7.80–7.83 (m, 2H, 1- H, 11-H), 7.92 (d, ^3J = 9 Hz, 1H, 12-H), 8.97 (s, 1H, 13-H), 9.96 (s, 1H, 8-H). – ^{13}C NMR (125 MHz, DMSO-d_6): δ = 26.3 (C5), 55.2 (C6), 55.6 (4’-OCH_3), 62.3 (9-OCH_3), 102.2 (OCH_2O), 105.7 (C1), 108.5 (C4), 115.4 (C2’, C6’), 119.4 (C3’, C5’), 120.0 (C13), 120.3 (C13b), 121.9 (C12a), 123.5 (C12), 131.1 (C4a), 131.7

(C11), 135.5 (C9), 139.0 (C13a), 145.7 (C8), 146.3 (C2), 146.6 (C8a), 147.8 (C10), 149.6 (C1’), 150.2 (C3), 155.9 (C4’). – MS (ESI$^+$): *m/z* (%) = 429 (100) [M-BF$_4$]$^+$. – El. Anal. for $C_{26}H_{22}BF_4NO_5$, calcd. (%): C 60.61, H 4.30, N 2.72, found (%): C 60.60, H 4.00, N 2.74.

9-Methoxy-10-(4-nitrophenoxy)-5,6-dihydro-[1,3]dioxolo[4,5-g]isoquinolino[3,2-a]isoquinolin-7-ium tetrafluoroborate (**5e**).

According to GP 2 the reaction of **4e** (20.0 mg, 46.7 µmol) with MeI (1.5 ml, 3.42 g, 24.1 mmol) and K_2CO_3 (6.45 mg, 46.7 µmol) gave product **5e** as yellow solid (19.1 mg, 36.0 µmol, 77%); mp > 269 °C (dec.). – ^{1}H NMR (500 MHz, DMSO-d_6): δ = 3.22 (t, ^3J = 6 Hz, 2H, 5-H), 4.10 (s, 3H, CH$_3$), 4.94 (t, ^3J = 6 Hz, 2H, 6-H), 6.20 (s, 2H, OCH$_2$O), 7.12 (s, 1H, 4-H), 7.27 (d, ^3J = 9 Hz, 2H, 2‘-H, 6‘-H), 7.85 (s, 1H, 1-H), 8.00 (d, ^3J = 9 Hz, 1H, 12-H), 8.08 (d, ^3J = 9 Hz, 1H, 11-H), 8.32 (d, ^3J = 9 Hz, 2H, 3´-H, 5´-H), 9.05 (s, 1H, 13-H), 10.02 (s, 1H, 8-H). – ^{13}C NMR (125 MHz, DMSO-d_6): δ = 26.2 (C5), 55.2 (C6), 62.6 (CH$_3$), 102.3 (OCH$_2$O), 105.8 (C1), 108.5 (C4), 117.2 (C2’, C6’), 120.1 (C13b), 120.4 (C13), 122.0 (C8a), 123.9 (C12), 126.4 (C3’, C5’), 131.4 (C4a), 133.6 (C11), 137.2 (C12a), 139.9 (C13a), 143.0 (C4’), 143.3 (C10), 146.1 (C8), 147.8 (C2), 148.1 (C9), 150.4 (C3), 161.7 (C1’). – MS (ESI$^+$): *m/z* (%) = 443 (100) [M-BF$_4$]$^+$. – El. Anal. for $C_{25}H_{19}BF_4N_2O_6$, calcd. (%): C 56.63, H 3.61, N 5.28, found (%): C 56.26, H 3.23, N 5.20.

Optimization of reaction parameters for the Chan-Evans-Lam coupling of berberrubine.

Table S1. Optimization of reaction parameters for the synthesis of derivative **4a**.

Entry[a]	Boronic acid	Solvent	Temperature	Base	Yield
1	**3a**	CH_2Cl_2	rt	NEt_3	5%
2	$NaPhBF_3$	CH_2Cl_2	rt	NEt_3	<3%
3	**3a**	MeOH	40 °C	NEt_3	<3%
4	**3a**	MeOH	40 °C	NEt_3	<3%
5	**3a**	DMF	rt	NEt_3	26%
6	**3a**	DMF	40 °C	NEt_3	19%
7	**3a**	DMF	rt	2,2'-Bipyridine	<3%
8	**3a**	DMF	rt	Phenanthroline	<3%
9	**3a**	DMF	rt	DMAP	<3%

[a] All reactions were performed with berberrubine (**1b**, 1.00 mmol), $Cu(OAc)_2$ (1.00 mmol), 4 molar equiv base and molecular sieves (4 Å) in 5 mL of the corresponding solvent. The reaction was stopped when TLC analysis indicated complete consumption of **1b**.

2. Photophysical properties

Solutions were prepared from stock solutions of the derivatives **5a–e** in MeOH (c = 1.0 mM). Aliquots of the stock solution were thoroughly evaporated under a stream of nitrogen, and the residue was redissolved in the respective solvent or solvent mixture.

Table S2: Absorption and emission properties of derivatives **5b**, **5c** and **5e** in different solvents.

Compound	Solvent	$\lambda_{abs}{}^{a}$ / nm	lg ε^{b}	$\lambda_{fl}{}^{c}$ / nm	$\Phi_{fl}{}^{d}$
**5b**	$CHCl_3$	420	3.88	532	0.03
	EtOH	416	3.87	561	<0.01
	MeOH	413	3.88	576	<0.01
	DMSO	410	3.83	[e]	[e]
	Buffer	410	3.81	[e]	[e]
**5c**	$CHCl_3$	421	3.85	531	0.03
	EtOH	415	3.85	559	<0.01
	MeOH	414	3.87	568	<0.01
	DMSO	410	3.82	[e]	[e]
	Buffer	410	3.64	[e]	[e]
**5e**	MeOH	408	3.76	[e]	[e]
	DMSO	405	3.79	[e]	[e]
	Buffer	403	3.78	[e]	[e]

[a] Long-wavelength absorption maximum; c_L = 20 µM. [b] ε = Molar extinction coeff. in cm^{-1} M^{-1}. [c] Fluorescence emission maximum; λ_{ex} = 415 nm. [d] Fluorescence quantum yield relative to coumarin 153 in EtOH (Φ_{fl} = 0.544) [1]. [e] Fluorescence quantum yield too low to be determined.

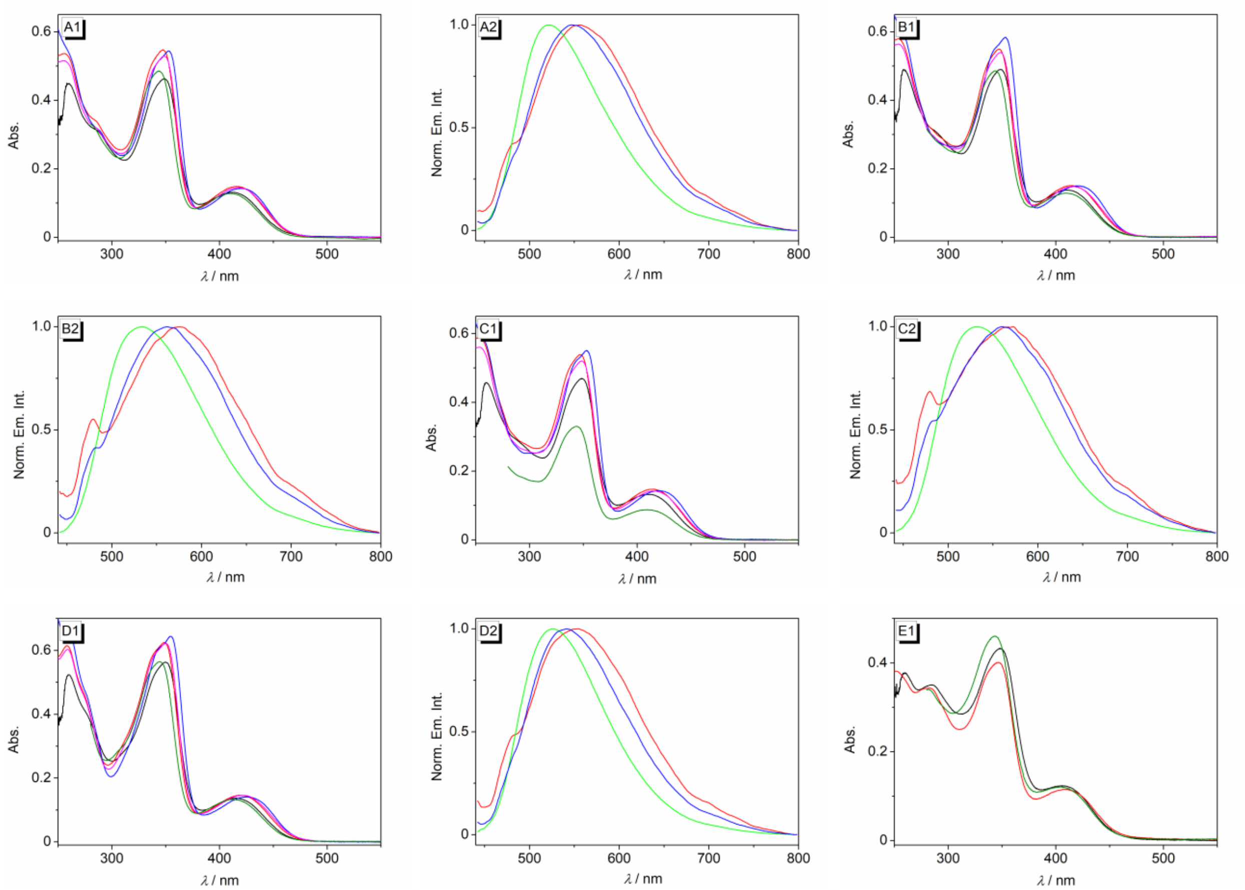

Figure S1. Absorption (1) and emission spectra (2) of **5a** (A), **5b** (B), **5c** (C), **5d** (D) and **5e** (E) (c = 20 µM) in different solvents (red: MeOH, black: DMSO, green: aq. buffer, magenta: EtOH, blue: $CHCl_3$; buffer: 16 mM BPE buffer solution at pH 7, λ_{ex} = 430 nm).

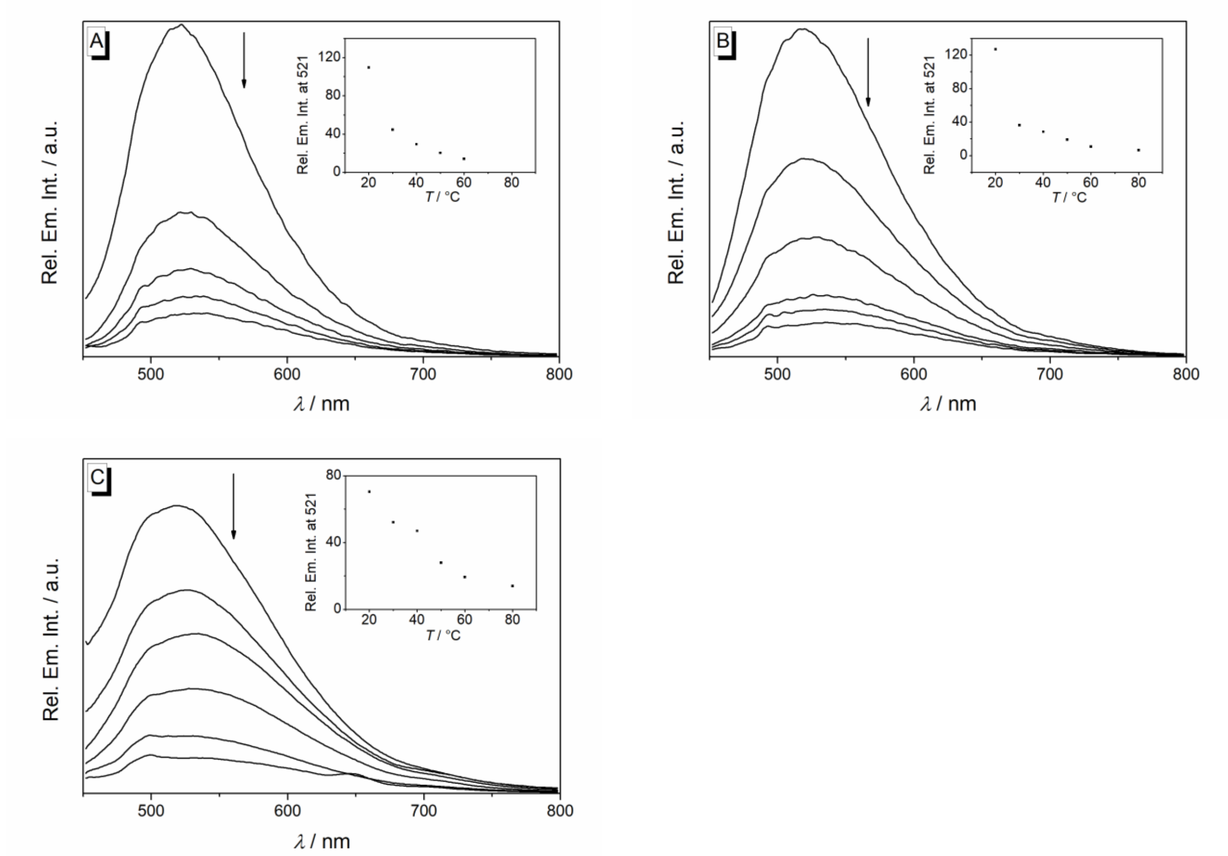

Figure S2. Emission spectra of derivatives **5b** (A), **5c** (B) and **5e** (C) (c = 10 µM, with 0.25% v/v DMSO) in glycerol; λ_{ex} = 430 nm. The arrows indicate the changes of the emission intensity with increasing temperature. Inset: Plot of the relative emission intensity *versus* the temperature of the solution.

3. Determination of the fluorescence quantum yields

Solutions of **5a**–**e** were prepared as described above from stock solutions in MeOH (c = 1.0 mM). The relative fluorescence quantum yields were determined under identical conditions (detection wavelength, excitation wavelength, detector voltage, slit bandwidths, collection rate). excitation and emission slits were adjusted to 5 nm, and the excitation wavelengths were fixed to 340 nm The quantum yield Φ_{fl} was determined according to equation 1. The estimated error is ca. 10% of the given values.

$$\Phi_{\mathrm{fl,\,X}} = \frac{F_X A_S}{F_S A_X} \cdot \frac{n_X^2}{n_S^2} \cdot \Phi_{\mathrm{fl,\,S}} \qquad \text{(eq. 1)}$$

The indices X and S indicate the analyte (X) and standard (S, coumarin 153 in ethanol, Φ_{fl} = 0.544 [1]) solution.

Φ_{fl} = Emission quantum yield.

F = Integral of the emission curve.

A = Absorbance at the excitation wavelength.

n = Refractive index.

4. Photometric and fluorimetric DNA titrations

Photometric and fluorimetric titrations with ct DNA and oligonucleotides **22AG** were performed according to published protocols [2]. All titrations were performed with 5% v/v DMSO to ensure a sufficient solubility of the ligand.

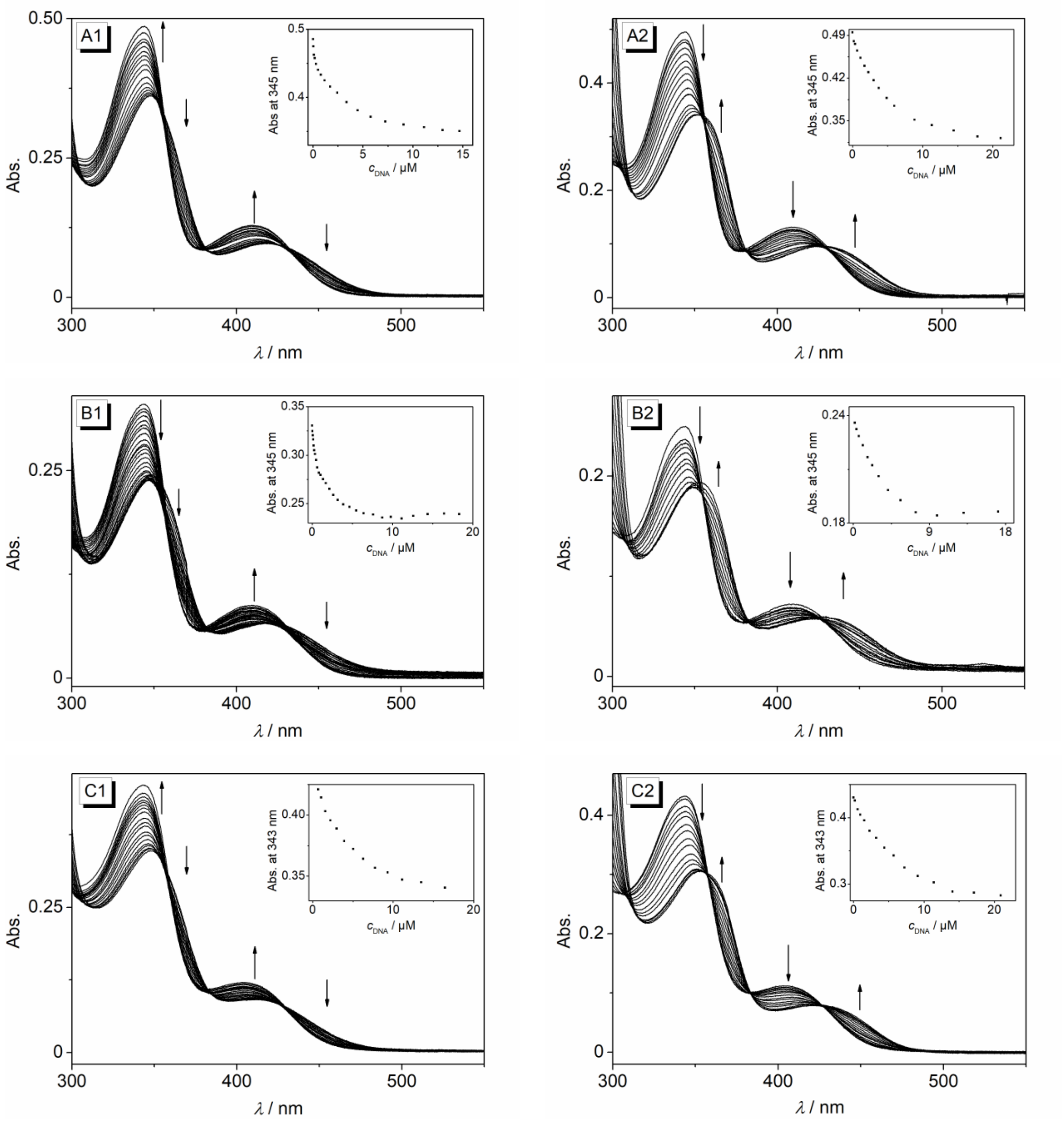

Figure S3. Photometric titration of **5b** (A), **5c** (B) and **5e** (C) (c_{Ligand} = 20 µM) with ct DNA (1) in BPE buffer (c_{Na+} = 16 mM, pH 7.0, with 5% v/v DMSO) and with **22AG** (2) in K-phosphate buffer (c_{K+} = 110 mM, pH 7.0, with 5% v/v DMSO). The arrows indicate the changes of the absorption bands upon addition of DNA. Inset: Plot of the ligand absorption versus c_{DNA} (in base pairs).

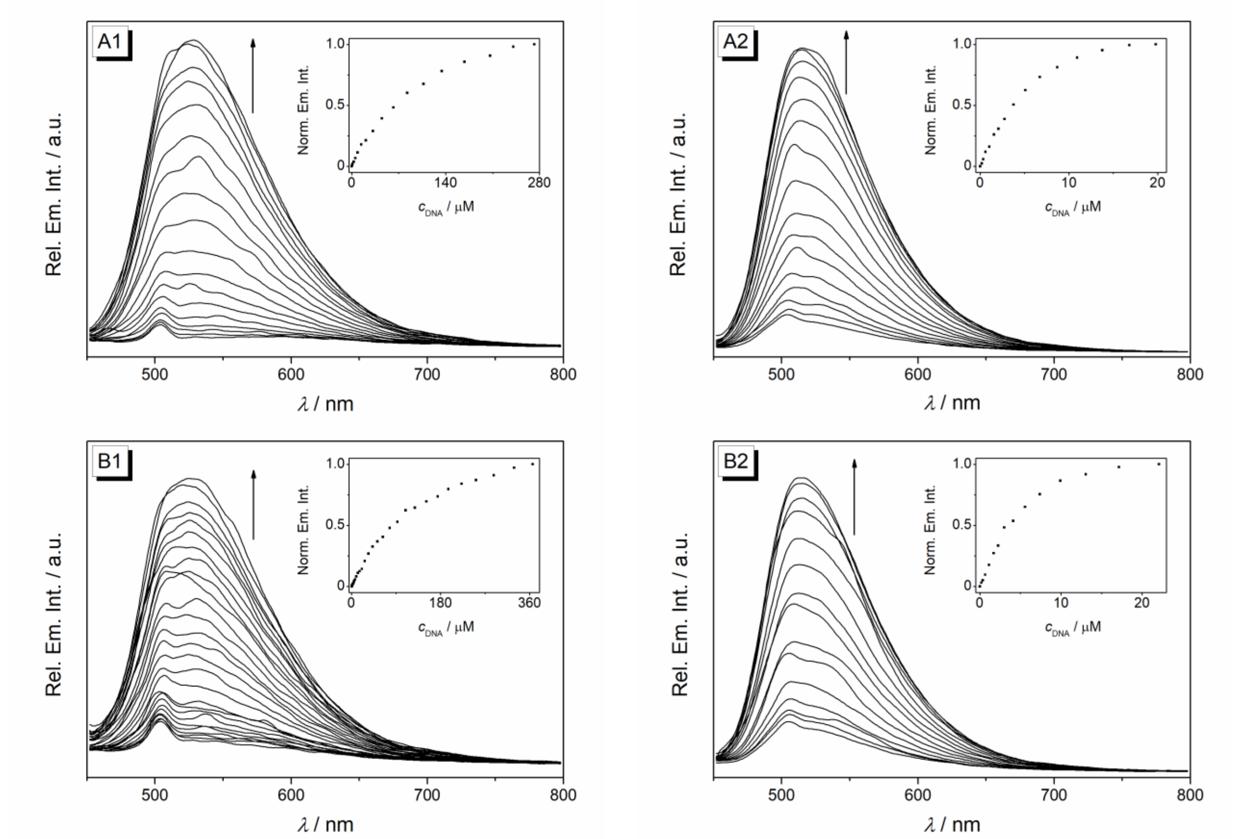

Figure S4. Fluorimetric titration of **5c** (A) and **5d** (B, c_{Ligand} = 20 µM) with ct DNA (1) in BPE buffer ($c_{\text{Na+}}$ = 16 mM, pH 7.0, with 5% v/v DMSO) and with **22AG** (2) in K-phosphate buffer ($c_{\text{K+}}$ = 110 mM, pH 7.0, with 5% v/v DMSO); λ_{ex} = 430 nm. The arrows indicate the changes of the emission bands upon addition of DNA. Inset: Plot of the relative fluorescence intensity versus c_{DNA} (in base pairs).

The binding constants, K_b, were determined from binding isotherms of the photometric titration spectra (Figure S5) and fitting of the experimental data to the theoretical model according to equation 2 [3].

$$\frac{I}{I_0} = 1 + \frac{Q-1}{2}\left(A + xn + 1 - \sqrt{(Q + xn + 1)^2 - 4xn}\right) \qquad \text{(eq. 2)}$$

$Q = I / I_0$ = Minimal absorbance in the presence of excess ligand

n = Number of independent binding sites per DNA

$A = 1 / (K_b \times c_{\text{Ligand}})$

$x = c_{\text{DNA}} / c_{\text{Ligand}}$ = Titration variable Ligand

Standard derivations (SD) of K_b values were calculated according to equation 3.

$$SD\ (K_b) = \{(SD\ of\ A\ /\ A)\ /\ A\}\ /\ c_{Ligand} \qquad (eq.\ 3)$$

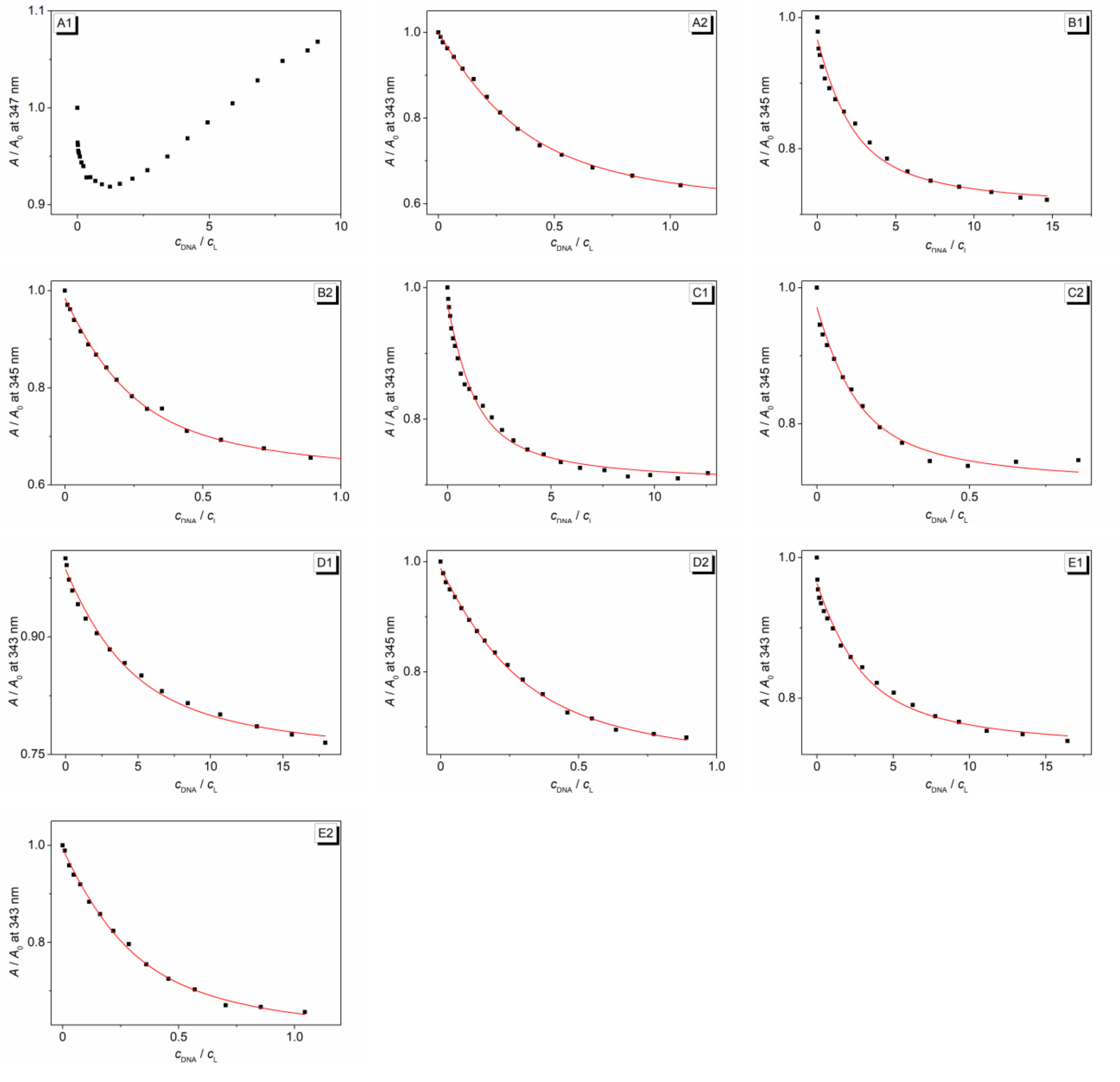

Figure S5. Fitting curves of binding isotherms resulting from spectrophotometric titrations of **5a** (A), **5b** (B), **5c** (C), **5d** (D) and **5e** (E) with ct DNA (1) and with **22AG** (2) for the determination of binding constants (K_b). Red lines represent the best fits of the experimental data to the theoretical model.

5. CD- and LD-spectroscopic analysis

CD and LD spectra were recorded in BPE buffer solution with ct DNA (c_{DNA} = 20.0 µM, 5% v/v DMSO) and in K-phosphate buffer solution with **22AG** (c_{DNA} = 20.0 µM, 5% v/v DMSO) at different ligand-DNA ratios (*LDR*). Measurements with ct DNA were performed at *LDR* = 0, 0.1, 0.2, 0.5, 1.0. CD signals were recorded with band width of 1 nm, recording speed of 1 nm/s and time per data point of 0.5 seconds.

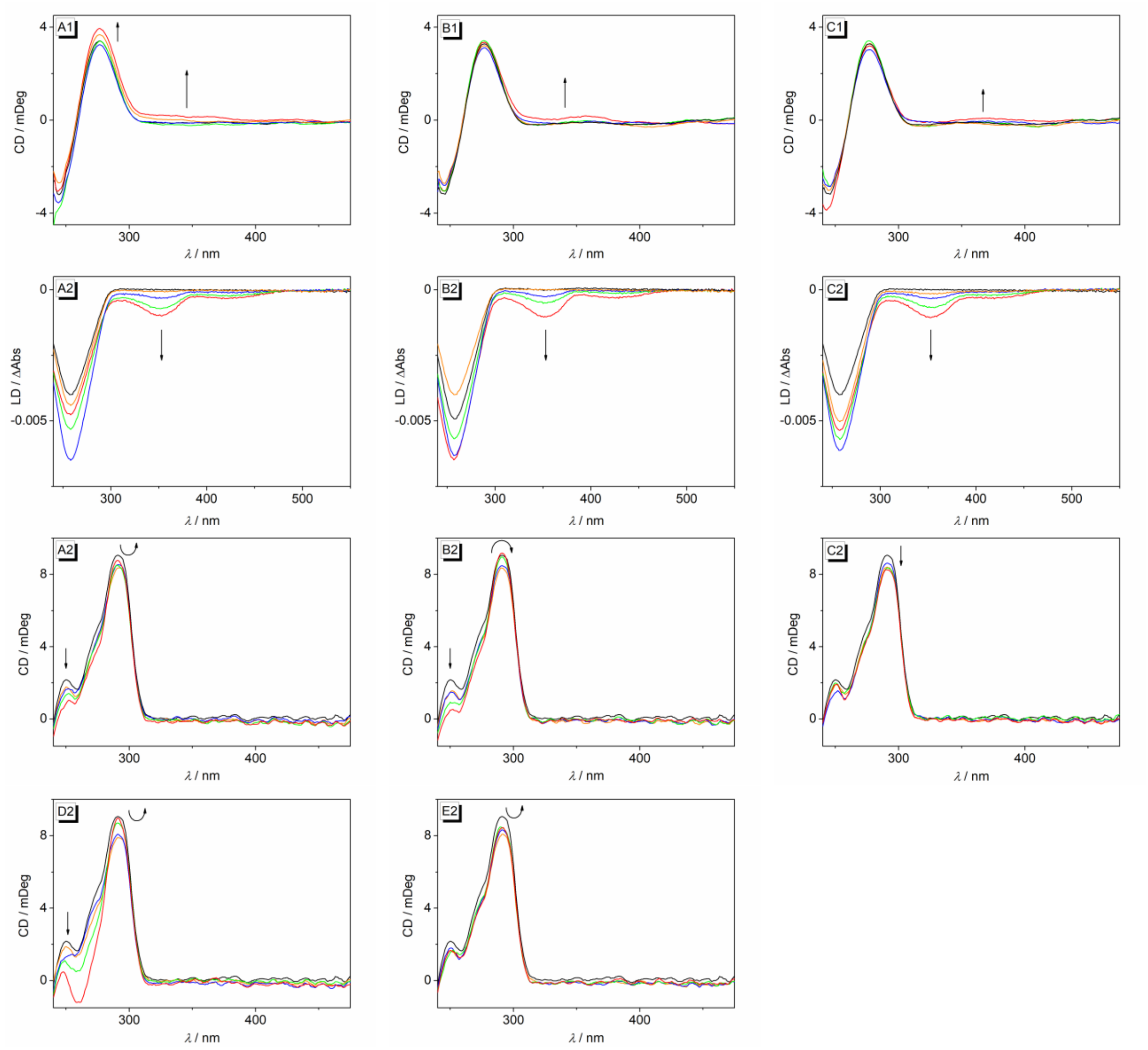

Figure S6. CD (1) and LD spectra (2) of ct DNA (*c* = 20 µM) in the absence and presence of **5a** (A), **5b** (B), **5c** (C), **5d** (D) and **5e** (E) in BPE buffer solution (10 mM, pH 7.0; with 5% v/v DMSO). CD spectra of **22AG** (3, *c* = 20 µM) in the absence and presence of **5a–e** in K-phosphate buffer (c_{K+} = 110 mM, pH 7.0, with 5% v/v DMSO) [LDR = 0 (black), 0.05 (orange), 0.2 (blue), 0.5 (green), 1.0 (red)]. The arrows indicate the changes of absorption on addition of DNA.

6. Thermal denaturation experiments

The stock solutions of the oligonucleotide **F21T**, **Fa2T**, **FmycT** and **FkitT** (c = 50 µM) and the ligand (c = 5 µM) were prepared in cacodylate buffer. The solution with the ligand contained 1% v/v DMSO. The samples were mixed according to Table S2. Melting temperatures, ΔT_m, were determined from the maximum of the first derivative of the melting curves. The determined T_m values before addition of ligand resembled the ones reported in literature [4–6].

Table S3. Composition of the samples for Fluorimetric DNA Denaturation Experiments with **F21T**, **Fa2T**, **FmycT** and **FkitT**.

Sample[a]	c_{Ligand} / µM	V_{Ligand} / µL[b]	V_{DMSO} / µL	V_{Puffer} / µL
1	0	0	10.0	996
2	0.25	50	9.50	971
3	0.50	100	9.00	946
4	1.00	200	8.00	896

[a] V_{DNA} = 4 µL c_{DNA} = 0.2 µM.[b] c_{DMSO} = 1 vol% c_{Ligand} = 10 µM.

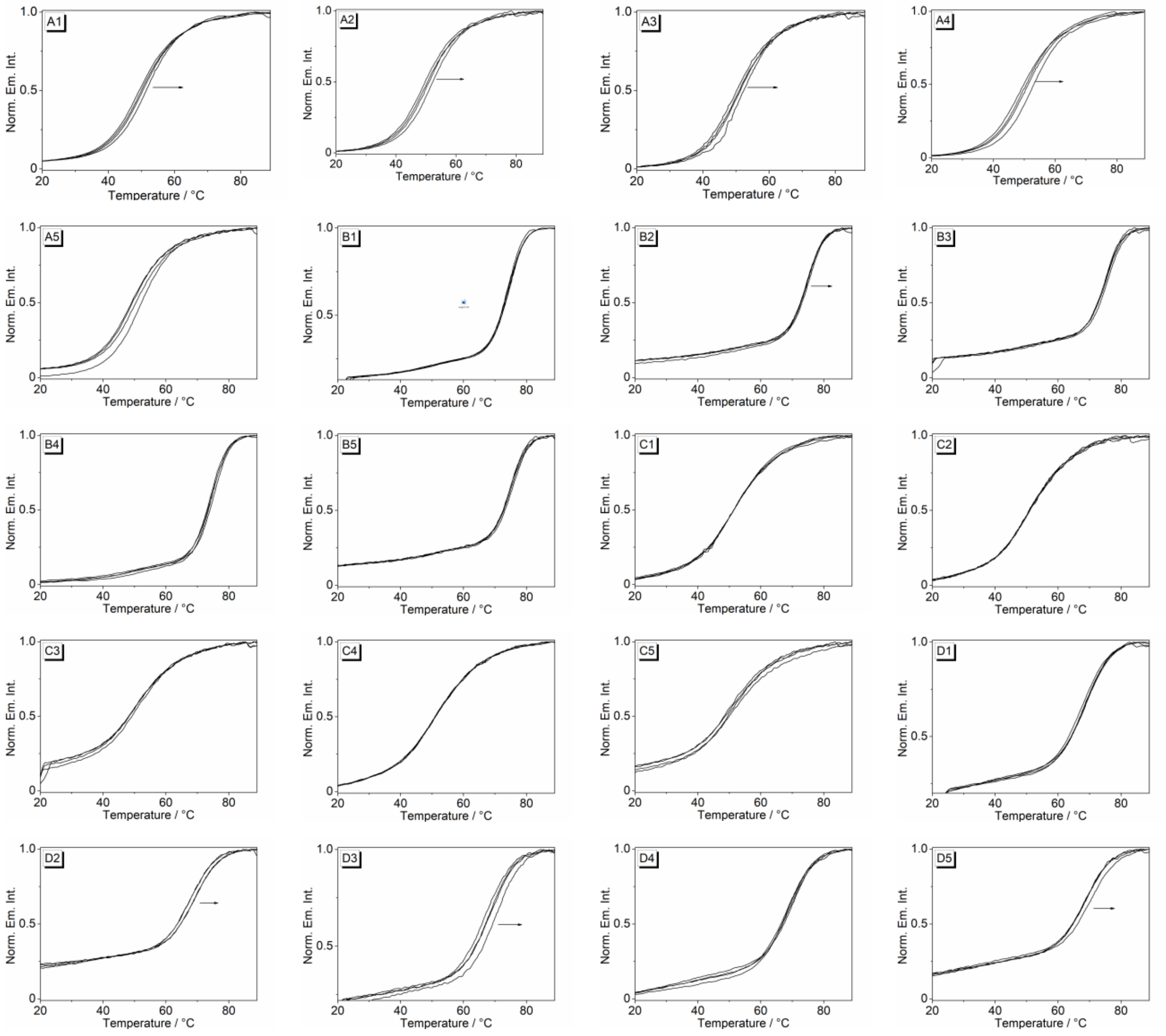

Figure S7. Normalized changes in emission intensity of with the oligonucleotides **F21T** (A), **Fa2T** (B), **FkitT** (C), **FmycT** (D) (0.2 µM, λ_{ex} = 470 nm) at 515 nm between 20 °C and 90 °C in presence of the ligands **5a** (1), **5b** (2), **5c** (3), **5d** (4) and **5e** (5) in aqueous KCl-LiCl-Na-cacolydate buffer (c_{K+} = 10 mM, c_{Na+} = 10 mM, c_{K+} = 90 mM, pH 7.2) at different ligand-DNA ratios (0, 1.3, 2.5, 5.0).

7. NMR spectra

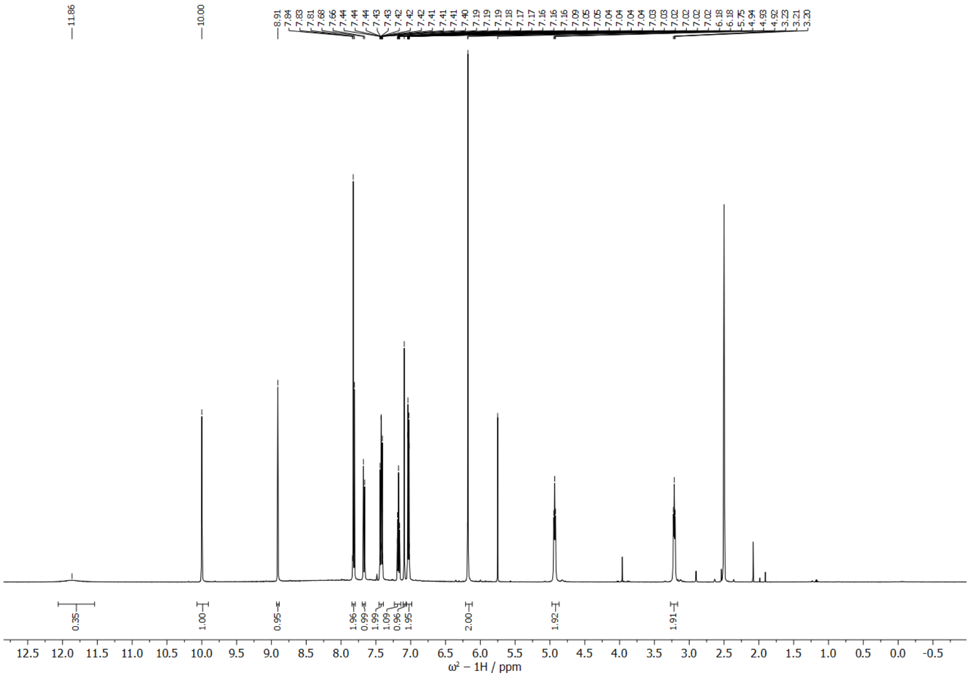

Figure S8. ^{1}H NMR spectrum (500 MHz) of **4a** in DMSO-d_6+ CF_3COOD.

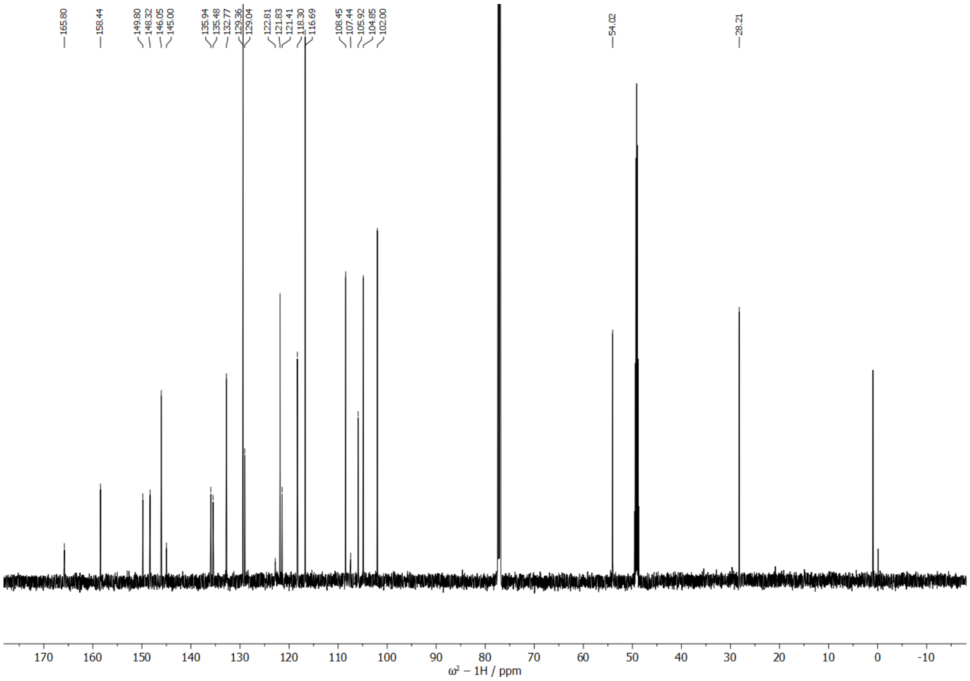

Figure S9. ^{13}C NMR spectrum (125 MHz) of **4a** in DMSO-d_6 + CF_3COOD.

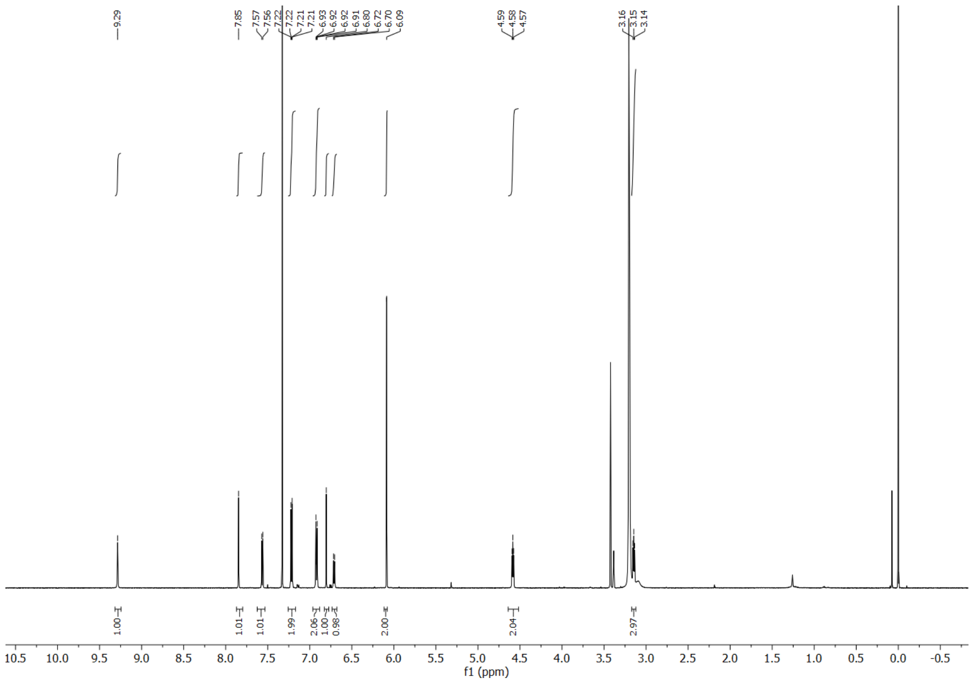

Figure S10. ^{1}H NMR spectrum (500 MHz) of **4b** in $CDCl_3$.

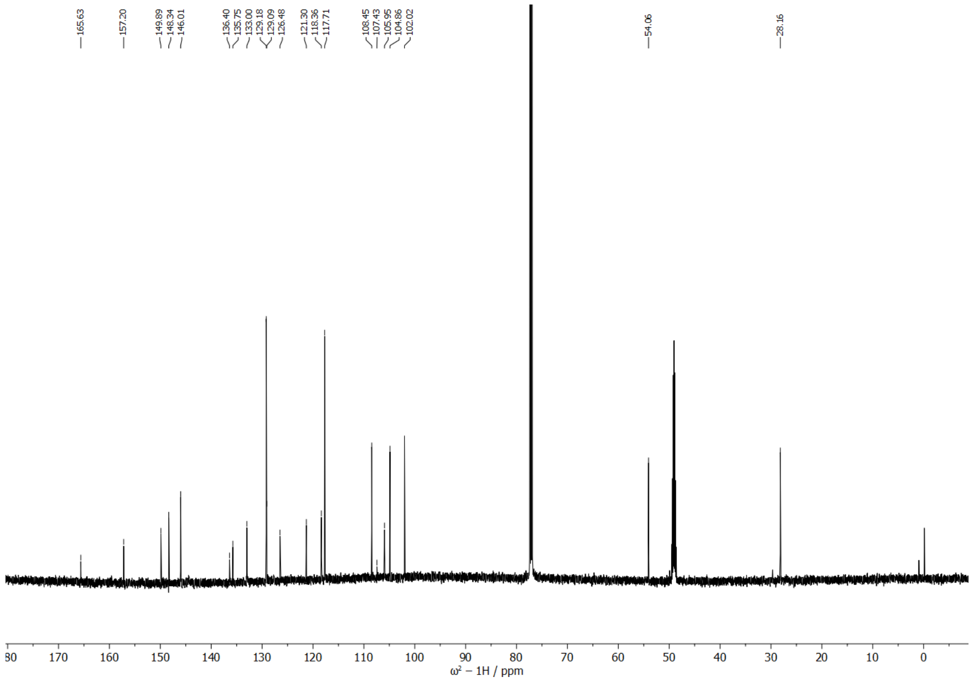

Figure S11. ^{13}C NMR spectrum (125 MHz) of **4b** in $CDCl_3$.

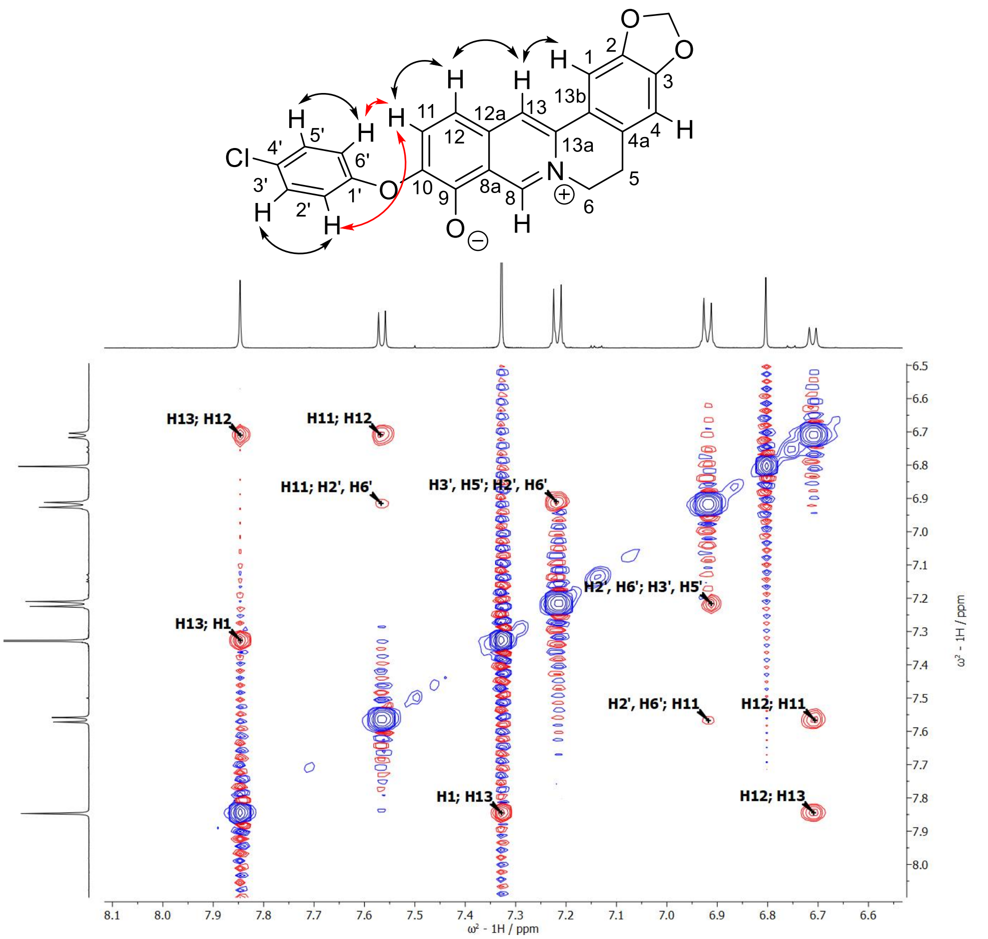

Figure S12. NOESY spectrum (500 MHz) of the aromatic region of **4b** in $CDCl_3$ and schematic representation of the NOE effects.

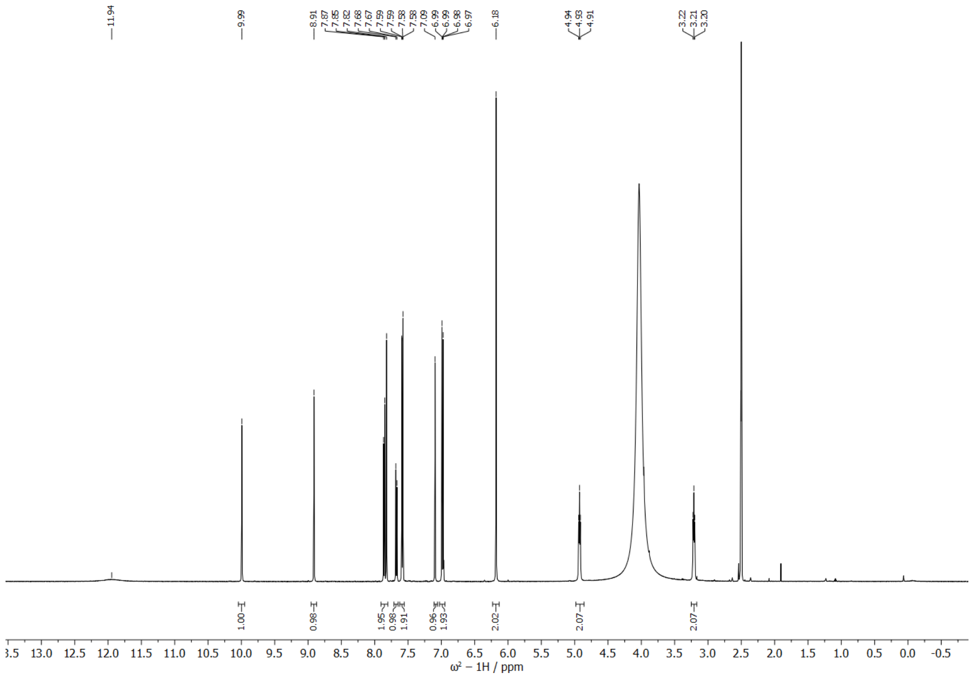

Figure S13. ^{1}H NMR spectrum (500 MHz) of **4c** in DMSO-d_6 + CF_3COOD.

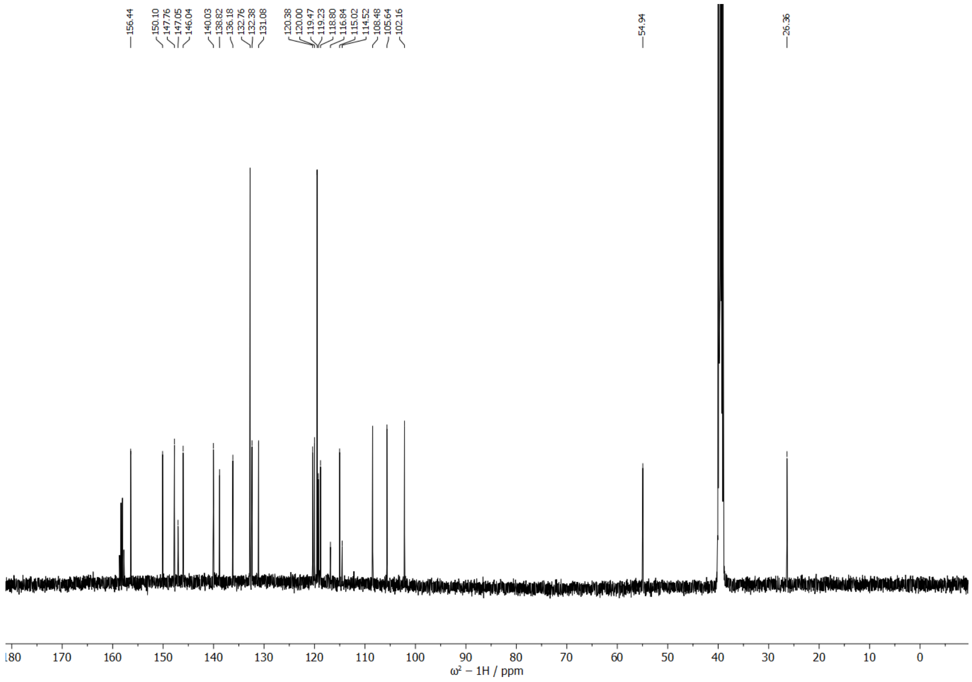

Figure S14. ^{13}C NMR spectrum (125 MHz) of **4c** in DMSO-d_6 + CF_3COOD.

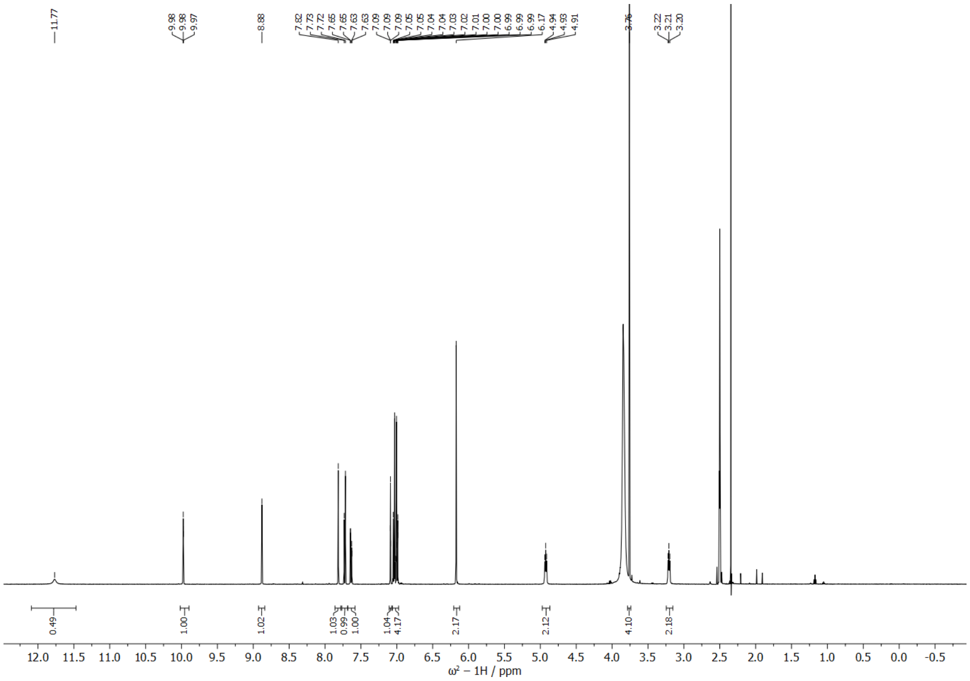

Figure S15. ^{1}H NMR spectrum (500 MHz) of **4d** in DMSO-d_6 + CF_3COOD.

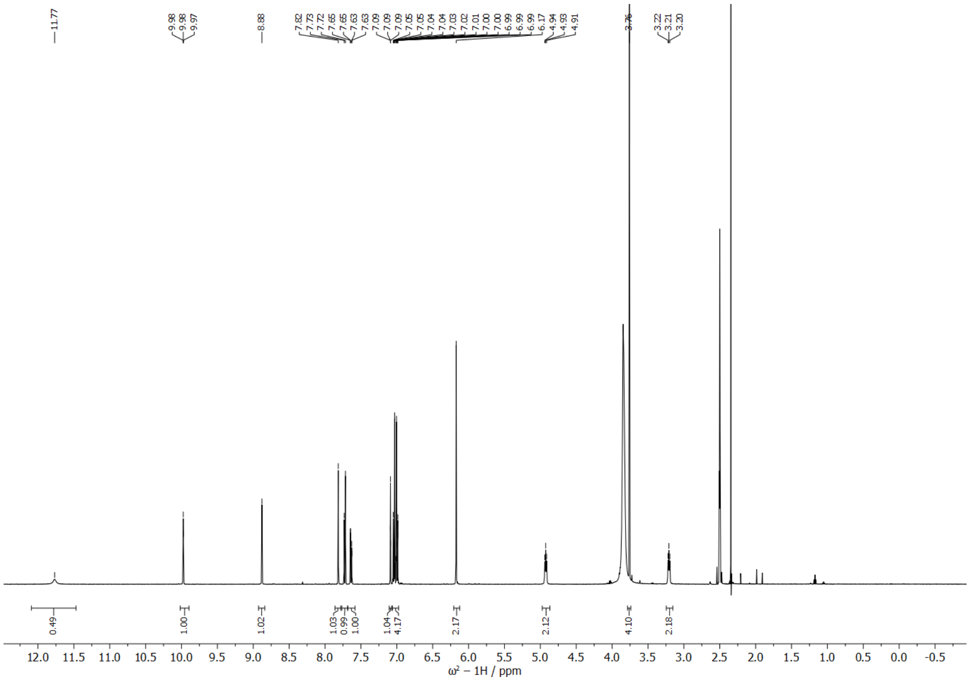

Figure S16. ^{13}C NMR spectrum (125 MHz) of **4d** in DMSO-d_6 + CF_3COOD.

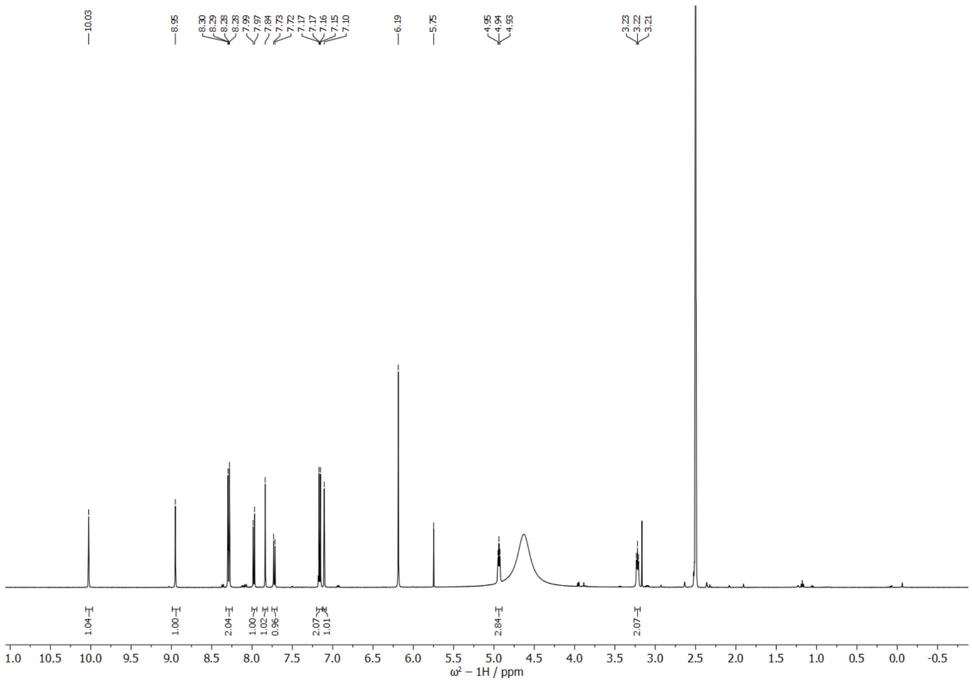

Figure S17. ^{1}H NMR spectrum (500 MHz) of **4e** in DMSO-d_6+ CF_3COOD.

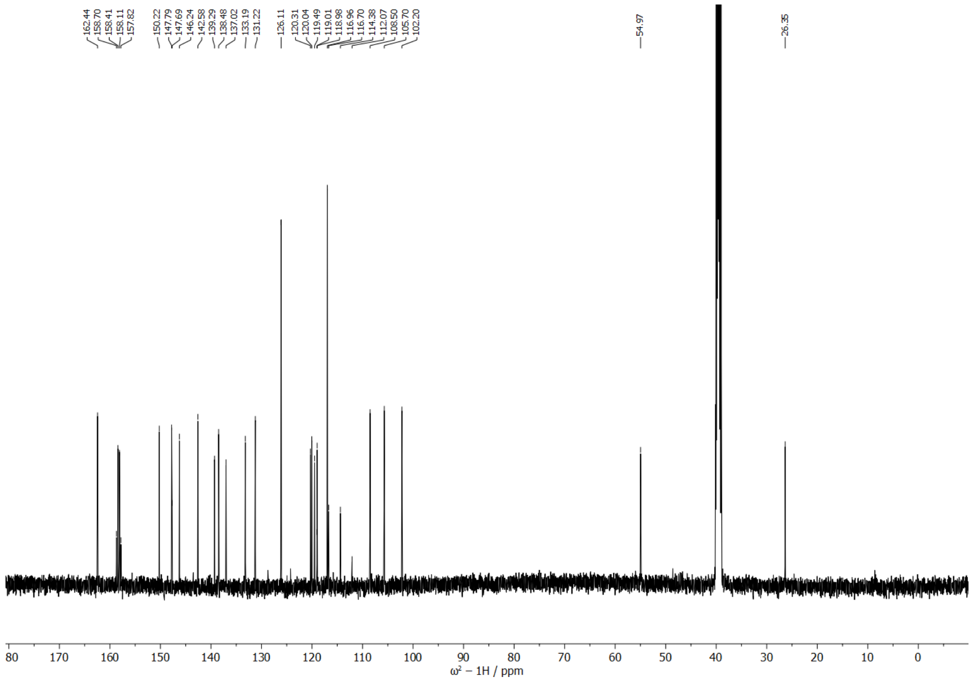

Figure S18. ^{13}C NMR spectrum (125 MHz) of **4e** in DMSO-d_6 + CF_3COOD.

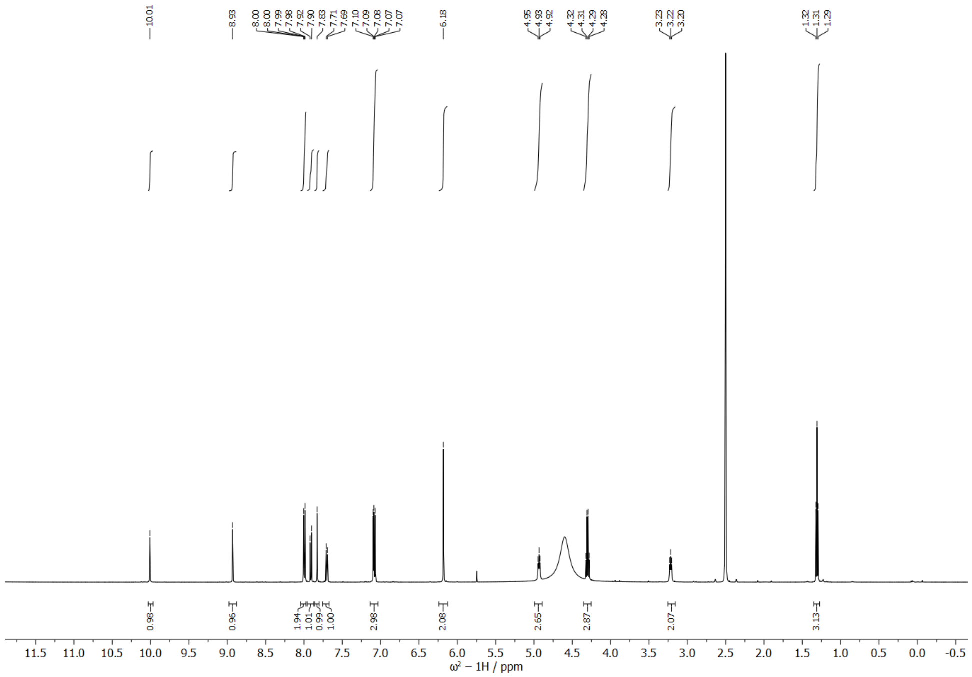

Figure S19. ^{1}H NMR spectrum (500 MHz) of **4f** in DMSO-d_6 + CF_3COOD.

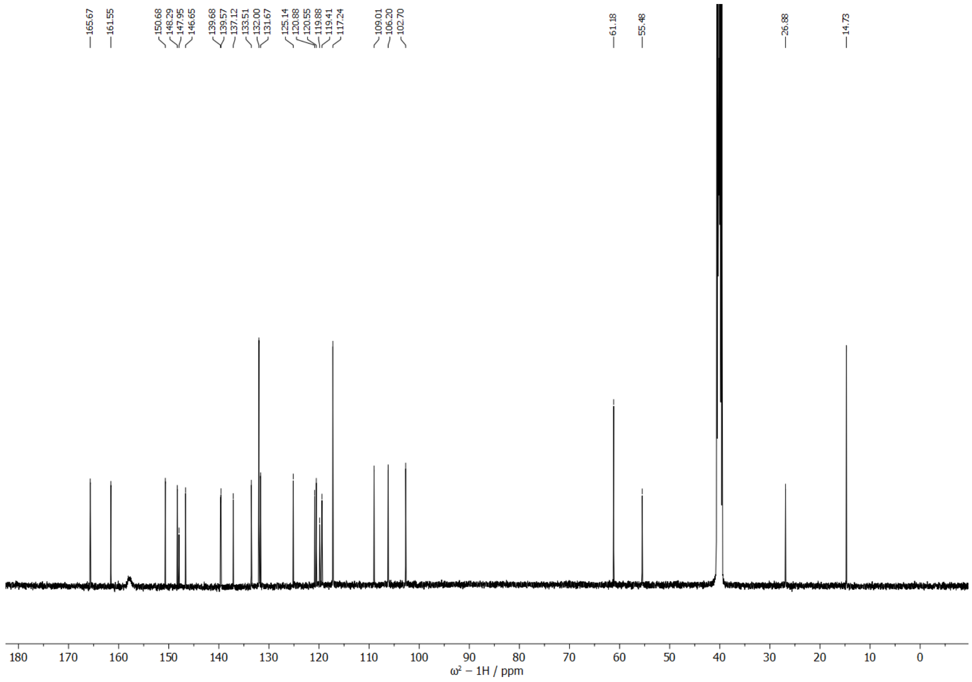

Figure S20. ^{13}C NMR spectrum (125 MHz) of **4f** in DMSO-d_6 + CF_3COOD.

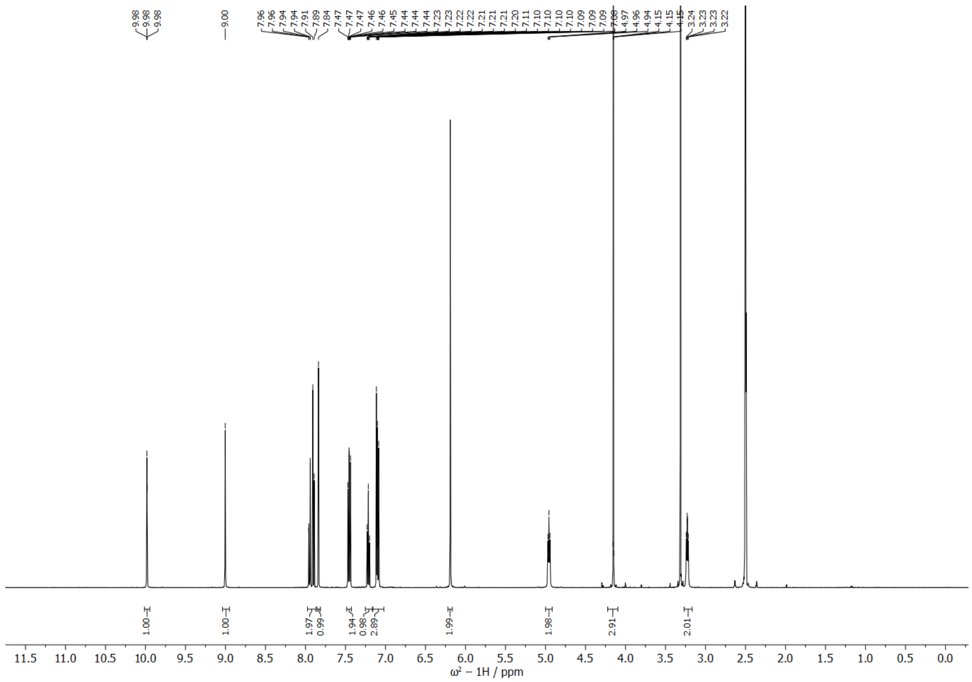

Figure S21. ^{1}H NMR spectrum (500 MHz) of **5a** in DMSO-d_6.

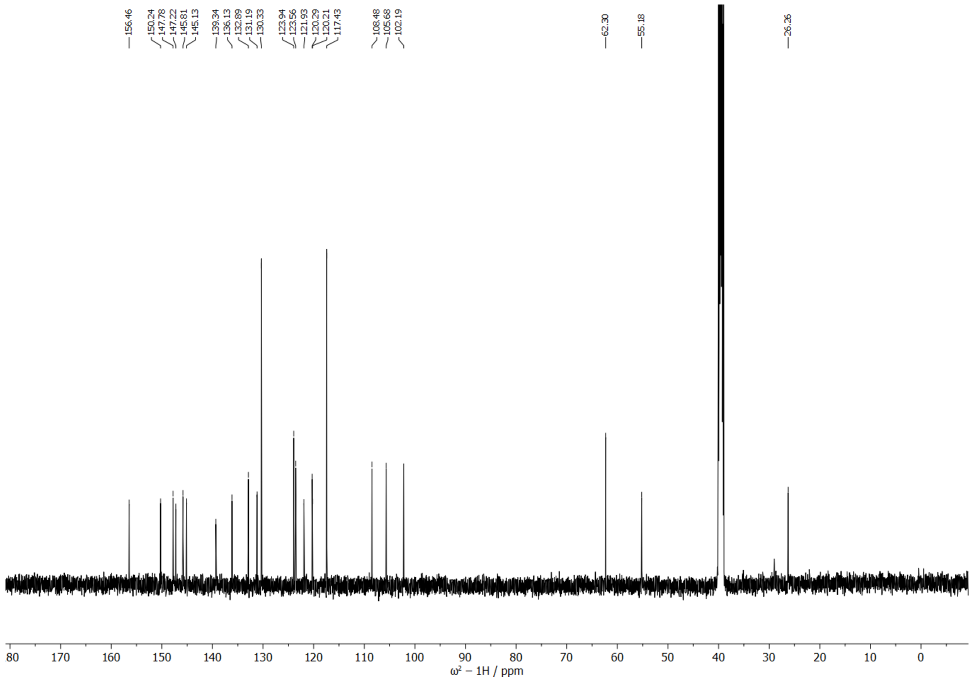

Figure S22. ^{13}C NMR spectrum (125 MHz) of **5a** in DMSO-d_6.

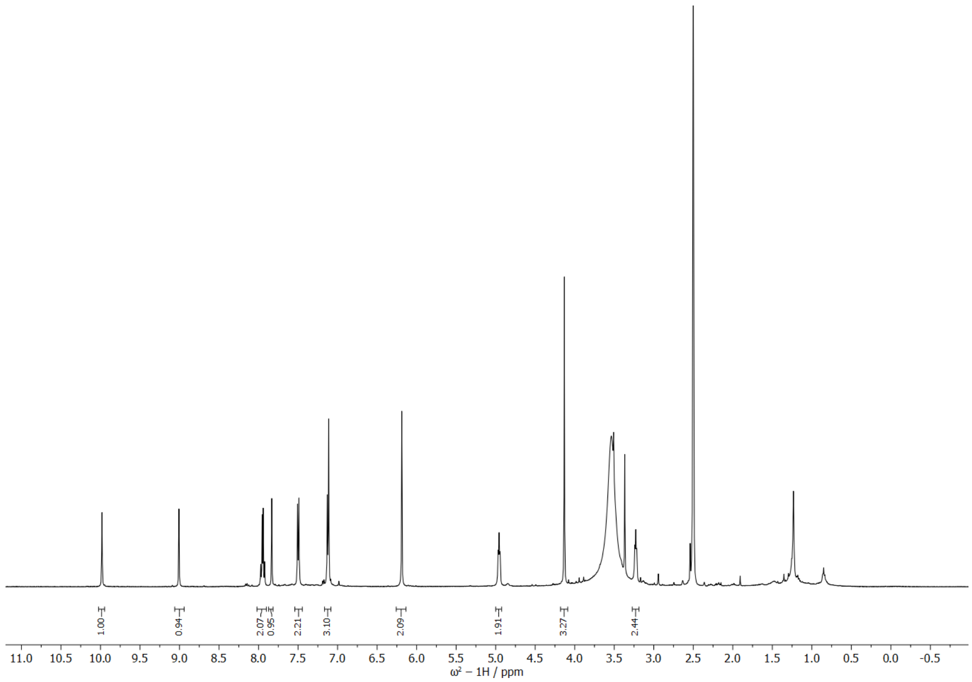

Figure S23. ^{1}H NMR spectrum (500 MHz) of **5b** in DMSO-d_6.

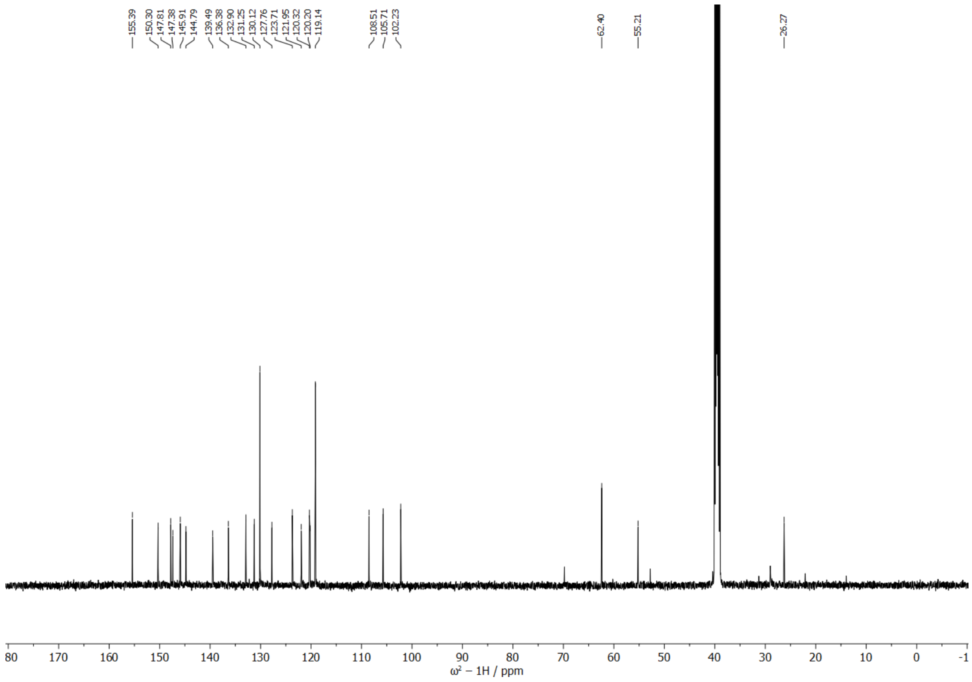

Figure S24. ^{13}C NMR spectrum (125 MHz) of **5b** in DMSO-d_6.

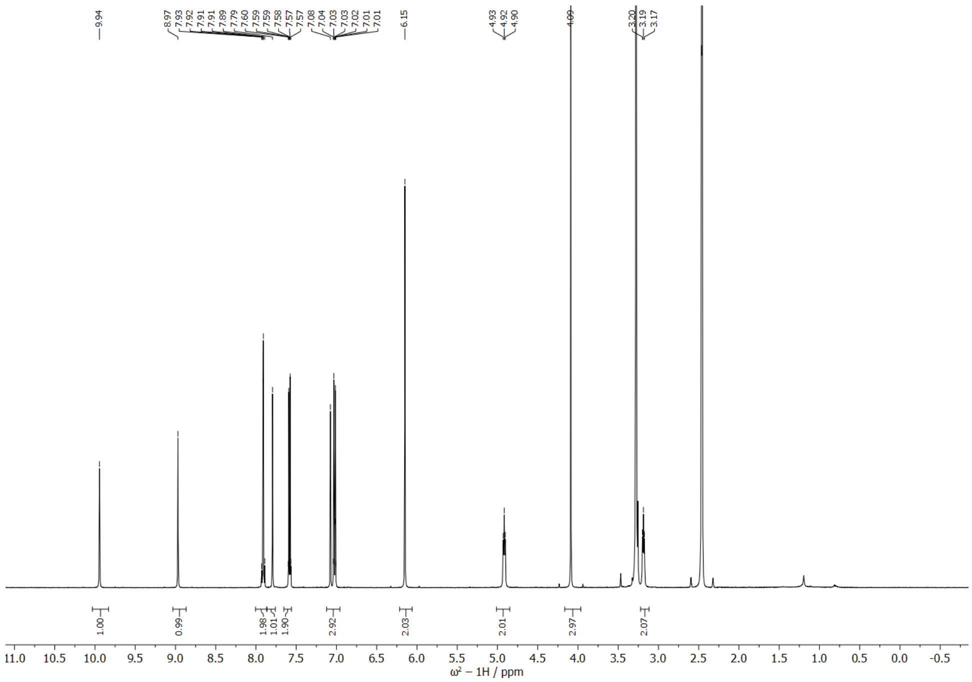

Figure S25. ^{1}H NMR spectrum (500 MHz) of **5c** in DMSO-d_6.

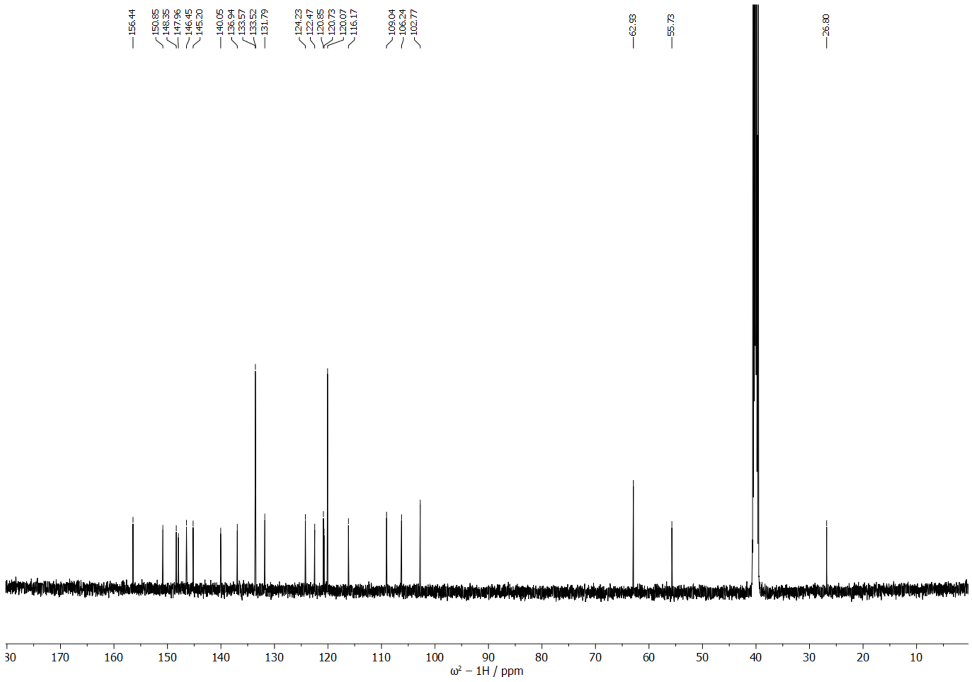

Figure S26. ^{13}C NMR spectrum (125 MHz) of **5c** in DMSO-d_6.

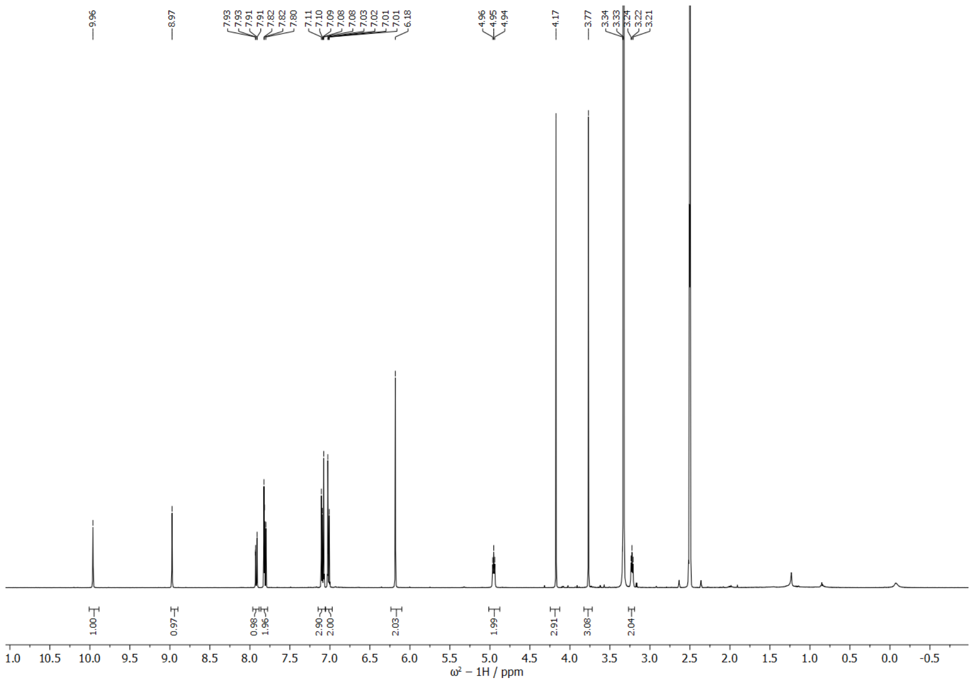

Figure S27. ^{1}H NMR spectrum (500 MHz) of **5d** in DMSO-d_6.

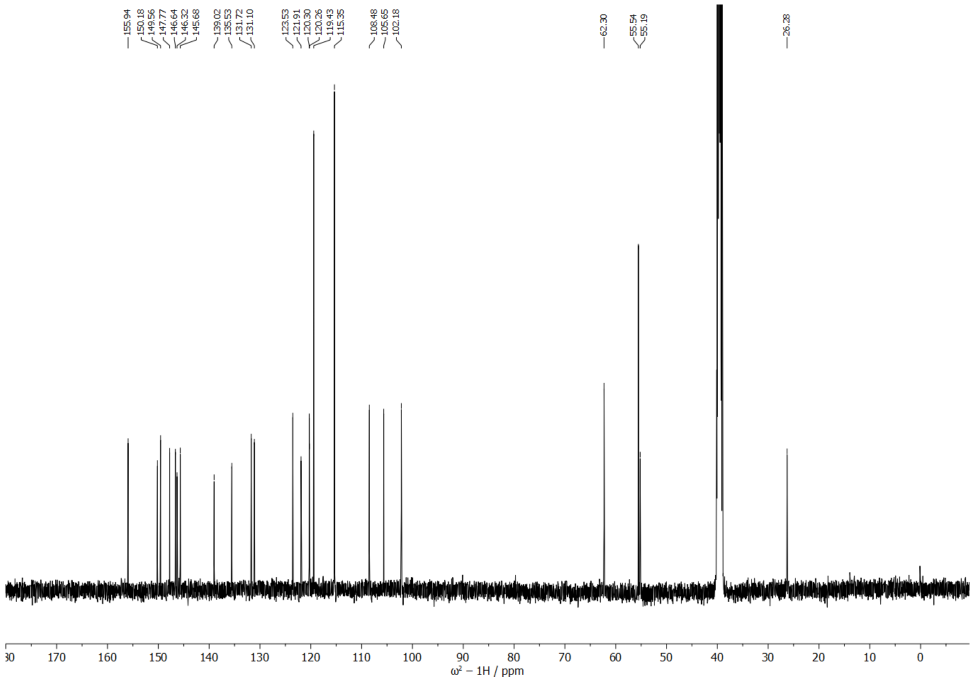

Figure S28. ^{13}C NMR spectrum (125 MHz) of **5d** in DMSO-d_6.

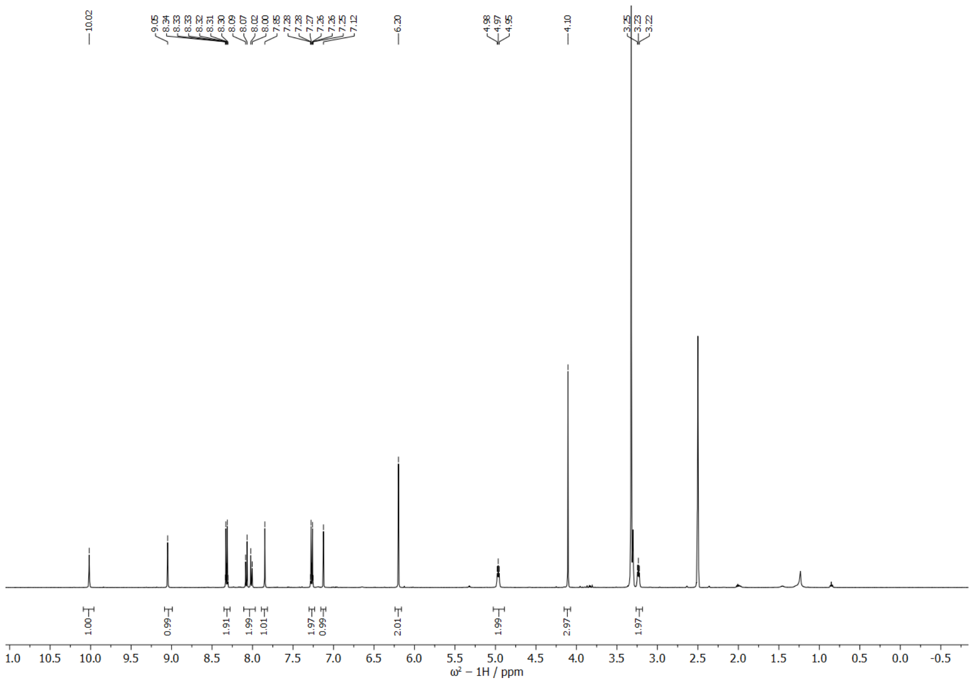

Figure S29. ^{1}H NMR spectrum (500 MHz) of **5e** in DMSO-d_6.

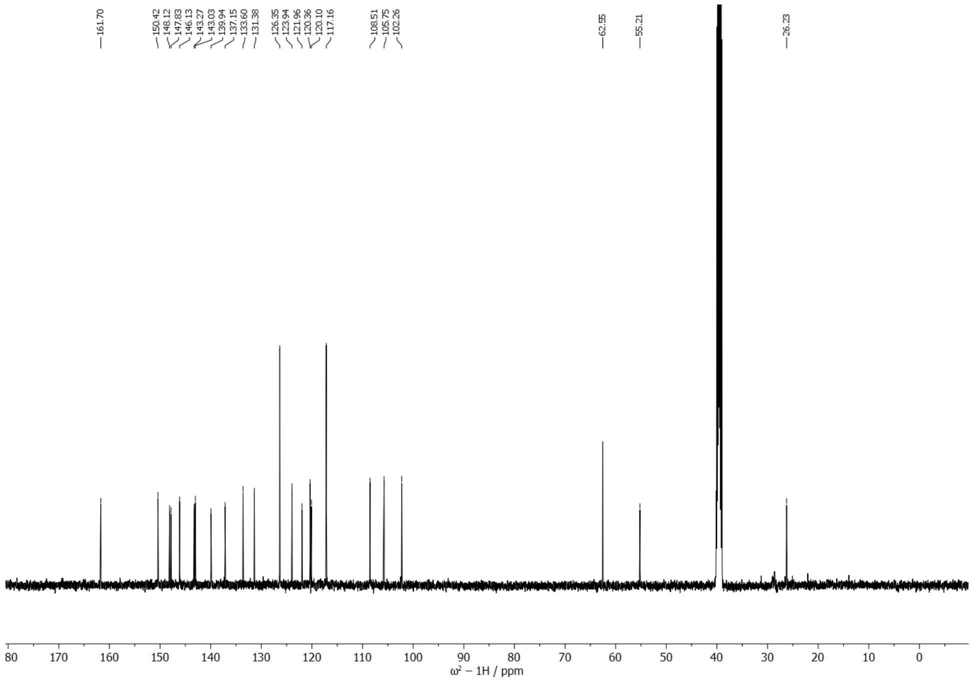

Figure S30. ^{13}C NMR spectrum (125 MHz) of **5e** in DMSO-d_6.

8. References

1. Rurack, K.; Spieles, M. *Anal. Chem.* **2011**, *83*, 1232–1242.

2. Bortolozzi, R.; Ihmels, H.; Thomas, L.; Tian, M.; Viola, G. *Chem. Eur. J.* **2013**, *19*, 8736–8741.

3. Stootman, F. H.; Fisher, D. M.; Rodger, A.; Aldrich-Wright, J. R. *Analyst* **2006**, *131*, 1145–1151.

4. Doria, F.; Nadai, M.; Costa, G.; Sattin, G., Gallati, C.; Bergamaschi, G.; Moraca, F.; Alcaro, S.; Freccero, M.; Richter, S. N. *Eur. J. Org. Chem.* **2016**, 4824–4833.

5. Dzubiel, D.; Ihmels, H.; Mohamed M. A. M.; Thomas, L. *Beilstein J. Org. Chem.* **2014**, *10*, 2963–2974.

6. Carvalho, J.; Quintela, T.; Gueddouda, N. M.; Bourdoncle, A.; Mergny, J.-L.; Salgado, G. F.; Queiroz, J. A.; Cruz, C. *Org. Biomol. Chem.* **2018**, *16*, 2776–2786.
